# Association between brain imaging signs, early and late outcomes, and response to intravenous alteplase after acute ischaemic stroke in the third International Stroke Trial (IST-3): secondary analysis of a randomised controlled trial

**DOI:** 10.1016/S1474-4422(15)00012-5

**Published:** 2015-05

**Authors:** The IST-3 collaborative group

## Abstract

**Background:**

Brain scans are essential to exclude haemorrhage in patients with suspected acute ischaemic stroke before treatment with alteplase. However, patients with early ischaemic signs could be at increased risk of haemorrhage after alteplase treatment, and little information is available about whether pre-existing structural signs, which are common in older patients, affect response to alteplase. We aimed to investigate the association between imaging signs on brain CT and outcomes after alteplase.

**Methods:**

IST-3 was a multicentre, randomised controlled trial of intravenous alteplase (0·9 mg/kg) versus control within 6 h of acute ischaemic stroke. The primary outcome was independence at 6 months (defined as an Oxford Handicap Scale [OHS] score of 0–2). 3035 patients were enrolled to IST-3 and underwent prerandomisation brain CT. Experts who were unaware of the random allocation assessed scans for early signs of ischaemia (tissue hypoattenuation, infarct extent, swelling, and hyperattenuated artery) and pre-existing signs (old infarct, leukoaraiosis, and atrophy). In this prespecified analysis, we assessed interactions between these imaging signs, symptomatic intracranial haemorrhage (a secondary outcome in IST-3) and independence at 6 months, and alteplase, adjusting for age, National Institutes of Health Stroke Scale (NIHSS) score, and time to randomisation. This trial is registered at ISRCTN.com, number ISRCTN25765518.

**Findings:**

3017 patients were assessed in this analysis, of whom 1507 were allocated alteplase and 1510 were assigned control. A reduction in independence was predicted by tissue hypoattenuation (odds ratio 0·66, 95% CI 0·55–0·81), large lesion (0·51, 0·38–0·68), swelling (0·59, 0·46–0·75), hyperattenuated artery (0·59, 0·47–0·75), atrophy (0·74, 0·59–0·94), and leukoaraiosis (0·72, 0·59–0·87). Symptomatic intracranial haemorrhage was predicted by old infarct (odds ratio 1·72, 95% CI 1·18–2·51), tissue hypoattenuation (1·54, 1·04–2·27), and hyperattenuated artery (1·54, 1·03–2·29). Some combinations of signs increased the absolute risk of symptomatic intracranial haemorrhage (eg, both old infarct and hyperattenuated artery, excess with alteplase 13·8%, 95% CI 6·9–20·7; both signs absent, excess 3·2%, 1·4–5·1). However, no imaging findings—individually or combined—modified the effect of alteplase on independence or symptomatic intracranial haemorrhage.

**Interpretation:**

Some early ischaemic and pre-existing signs were associated with reduced independence at 6 months and increased symptomatic intracranial haemorrhage. Although no interaction was noted between brain imaging signs and effects of alteplase on these outcomes, some combinations of signs increased some absolute risks. Pre-existing signs should be considered, in addition to early ischaemic signs, during the assessment of patients with acute ischaemic stroke.

**Funding:**

UK Medical Research Council, Health Foundation UK, Stroke Association UK, Chest Heart Stroke Scotland, Scottish Funding Council SINAPSE Collaboration, and multiple governmental and philanthropic national funders.

## Introduction

Prompt treatment with intravenous alteplase improves independent survival after acute ischaemic stroke.^1,2^ However, concerns about the risk of symptomatic intracranial haemorrhage and whether patients with early signs of ischaemia on CT should receive alteplase could be deterring use of this treatment.^3^

Early signs of ischaemia on non-enhanced brain CT include tissue hypoattenuation, lesion swelling, and arterial hyperattenuation from occlusive thrombus. Tissue hypoattenuation might represent irreversible tissue damage^4^ and has been associated with increased risk of symptomatic intracranial haemorrhage in some,^5^ but not all,^6,7^ studies. These signs are rarely seen alone, but the association between combinations of signs and prognosis after stroke or their interaction with alteplase is unknown. Moreover, in some alteplase trials, patients with specific findings (eg, extensive tissue hypoattenuation) were excluded.^5,8^

Other pathological features—such as leukoaraiosis, cerebral atrophy, and old infarcts—are often seen on brain CT in patients with acute stroke. These pre-existing signs might indicate brain frailty^9,10^ (ie, a vulnerability to ischaemia) or increased risk of symptomatic intracranial haemorrhage.^11^ The association of pre-existing signs with response to alteplase, with or without early ischaemic signs, is unknown.

The third International Stroke Trial (IST-3)^12^ is the largest (n=3035) randomised controlled trial of alteplase in acute stroke to date^1,2^ and was designed to test whether a wider range of patients could benefit from alteplase up to 6 h after stroke. The primary endpoint of IST-3—an increase in independent survival (defined as an Oxford Handicap Scale [OHS] score of 0–2 at 6 months)—did not differ between alteplase and control groups (odds ratio 1·13, 95% CI 0·95–1·35; p=0·181);^12^ however, in prespecified secondary analyses (ordinal shift analysis of the OHS and an OHS score of 0–1), alteplase increased functional outcome in all patients randomised up to 6 h and increased the primary outcome in the patients treated less than 3 h after stroke. The effect of alteplase 18 months after stroke has been published elsewhere.^13^ The findings of IST-3 accord with those of previous alteplase trials, at all time windows.^2^

A secondary objective of IST-3 was to ascertain whether early ischaemic or pre-existing brain CT signs—individually or in combination—were associated with response to alteplase on several clinically relevant early and late outcomes.^14^ Here, we present that secondary analysis.

## Methods

### Study design and participants

IST-3 was an international, multicentre, open-label, prospective blinded endpoint, randomised controlled trial done at 156 centres in 12 countries (appendix pp 2–5). Patients were eligible for the study if the treating clinician felt there was no clear indication for or contraindication to alteplase and judged the treatment promising but unproven for that individual. Eligibility criteria were age 18 years or older (no upper age limit) and symptoms of cortical, lacunar, and posterior circulation stroke,^15^ of all severities, according to the National Institutes of Health Stroke Scale (NIHSS). All participants had to be able to undergo randomisation and start treatment within 6 h of stroke. We did not exclude patients with early ischaemic or pre-existing imaging signs on brain CT. We excluded individuals younger than 18 years, people with standard contraindications to alteplase, patients with established visible infarction (ie, indicating stroke onset was likely to be more than 6 h previously), and individuals with haemorrhagic stroke or non-stroke lesion as the cause of stroke.

The IST-3 protocol^16^ and statistical analysis plan^14^ have been published elsewhere. The study was approved by the Scotland A research ethics committee and by ethics committees and other regulatory bodies of all participating countries, hospitals, and institutions. All patients, or a relative if the patient lacked capacity, provided written informed consent before randomisation into the trial.

### Randomisation and masking

Full details of the IST-3 randomisation procedure, including the minimisation process, are presented elsewhere.^12,16^ Briefly, the randomising clinician recorded baseline data via a central, secure, telephone-based or web-based system. The system randomly allocated patients to either standard best medical care plus immediate thrombolysis with intravenous alteplase (0·9 mg/kg bodyweight; maximum 90 mg; 10% bolus with the remainder over 1 h) or control (standard best medical care alone) within 6 h of ischaemic stroke. Patients allocated to the control group received the same stroke care as did individuals allocated to the alteplase group. The first 276 patients enrolled to the study were treated in a placebo-controlled, double-blind phase (the placebo was an inert product identical in appearance to alteplase); thereafter, patients were randomised to either immediate alteplase or open control.

### Procedures

To be approved for inclusion in IST-3, all participating centres had to pass minimum image acquisition standards^16^ to ensure that brain scans were of diagnostic quality for acute stroke (for CT) and included the minimum correct sequences (for MRI). Before randomisation, all patients had either a CT or MRI brain scan, repeated 24–48 h after stroke and again if any neurological deterioration arose in the first 7 days. The present analysis concerns brain images taken before randomisation, up to 6 h after stroke.

Participating centres sent all brain images to the IST-3 coordinating centre for adjudication and quality checking; all scans were anonymised. Adjudicators were masked to all information, except whether the scan was prerandomisation or follow-up. They viewed the images and recorded their ratings on a secure web-based system (systematic image review system [SIRS]). All adjudicators were neuroradiologists (JMW, RvK, AvH, LC, NB, ZM, AF, GP) or stroke neurologists (AP, AA) with experience in stroke imaging; they rated 60 scans from the ACCESS study^17,18^ and 25 scans from IST-3 that were selected at random, to ensure satisfactory agreement (defined as κ>0·70) for presence of early ischaemic signs and no more than one category difference for pre-existing signs. Adjudicators completed all analyses before the database was locked and the randomisation code was broken. Every scan was read by one adjudicator. Any discrepancies between the scan rating and clinical data (eg, side of brain affected, presence of haemorrhage) identified during data cleaning before data lock were cross-checked by JMW, who assigned a final rating before unmasking took place and the randomisation code was broken.

We first checked brain CTs for early ischaemic signs (tissue hypoattenuation, lesion size, swelling, and hyperattenuated artery); we used the term visible infarct to represent any of these signs. We then looked for pre-existing structural signs (old infarcts, leukoaraiosis, and atrophy).^19^ We classified images using validated scores.^17,18^ We classed scans as normal only if no early ischaemic changes or pre-existing changes were present.

We defined the presence and degree of hypoattenuated tissue as either mild (grey matter attenuation equal to normal white matter) or severe (grey and white matter attenuation slightly less than normal white matter but still consistent with onset of stroke within 6 h).^20^ We classified the extent of acute ischaemic lesions in three ways: with the one-third middle cerebral artery (MCA) method;^21,22^ with the IST-3 method;^17,23^ and with the Alberta Stroke Program Early CT Stroke (ASPECTS) score.^24^ The IST-3 score for infarct extent reflects all arterial territories, whereas the ASPECTS score and one-third MCA method focus only on the MCA territory. Therefore, we used the IST-3 score as the primary measure of infarct size in analyses, condensing the full IST-3 lesion extent score into four groups for analysis: small infarcts, which we defined as lacunar, small cortical, small cerebellar, less than half of brainstem, or less than half of the anterior cerebral artery (ACA) or posterior cerebral artery (PCA) territory; medium infarcts, classed as striatocapsular, the anterior or posterior half of the peripheral MCA territory, or more than half the ACA or PCA territory; large infarcts, defined as the whole of the peripheral MCA territory or all the MCA territory; and very large infarcts, which comprised the whole MCA and PCA territory, all the MCA and ACA territory, or all three territories.^24^ Small infarcts on the IST-3 score were equivalent to ASPECTS 8–10, medium infarcts corresponded to ASPECTS 5–7, and large and very large infarcts were similar to ASPECTS 0–4. MCA involvement in small and medium infarcts (according to IST-3 score) was equivalent to less than one-third MCA, and in large and very large infarcts it corresponded to more than one-third MCA. We graded ischaemic lesion swelling on a seven-point scale.^23^ We noted the presence or absence and location of any hyperattenuated artery.^17,25^

We recorded the location of old infarcts (eg, cortical, lacunar, border zone, and brainstem or cerebellar).^23^ We noted the presence and severity of leukoaraiosis on CT;^26^ if brain MRI was done, we used the Fazekas scale to grade changes.^27^ We classified atrophy as none, moderate, or severe when compared against standard examples. We also classed follow-up scans for any haemorrhage (petechial, haematoma in or remote from infarct, intraventricular, or subarachnoid blood), including whether the haemorrhage was likely to worsen neurological status, as part of the outcome assessment for symptomatic intracranial haemorrhage.

### Outcomes

The primary outcome of IST-3 was the proportion of patients alive and independent, defined by an OHS score of 0–2, at 6 months. The OHS^28^ is similar to the modified Rankin scale^29,30^ and has values from 0 to 6, with 0 representing no symptoms or completely independent and 6 signifying the patient has died. OHS score 0–2 indicates good functional outcome. We also analysed two prespecified secondary endpoints:^14^ an ordinal analysis of the OHS; and OHS score of 0–1 (ie, favourable outcome). Other secondary outcomes were symptomatic intracranial haemorrhage within 7 days,^14^ death within 7 days, and death by 6 months.

Local staff at every centre followed up all patients for 7 days and recorded early outcomes (symptomatic intracranial haemorrhage, death, and likely cause of death before 7 days), which were sent to the central trials office. At 6-month follow-up, central researchers who were masked to group allocations sent questionnaires by post to patients or their carers, to record independence (using the OHS);^16^ if we knew the patient had died, we sent the questionnaire to their family doctor, to obtain the date of death and likely cause.

### Statistical analyses

Analyses of early ischaemic and pre-existing structural brain signs, their effect on functional outcome, death, and symptomatic intracranial haemorrhage, and interactions with alteplase, were prespecified in the trial protocol and analysis plan.^14^ We analysed imaging data for patients whose prerandomisation scans were received at the central trials office. We did statistical analyses with SAS version 9.3.

We used logistic regression to ascertain associations between imaging signs and: (a) age, NIHSS score, and time to randomisation; and (b) outcomes at 7 days (symptomatic intracranial haemorrhage) and 6 months (OHS score of 0–2 and OHS score of 0–1), which were judged representative of the most clinically relevant early hazard and late benefit. We did multivariate logistic regression, adjusting for the linear effects of age, NIHSS score, and time to randomisation, to identify whether any combinations of imaging variables were associated with symptomatic intracranial haemorrhage or OHS score of 0–2. We then tested for interactions of imaging signs (presence, absence, or severity) and response to alteplase (adjusting for age, NIHSS score, and time to randomisation) with symptomatic intracranial haemorrhage, death within 7 days, and OHS score of 0–2 at 6 months; as a secondary analysis, we assessed interactions of imaging signs and response to alteplase with an OHS score of 0–1 and by ordinal OHS at 6 months (a more statistically sensitive ordinal model than analysis of dichotomous OHS categories). We tested whether associations between imaging signs and outcomes and imaging signs and alteplase differed in patients randomised within 3 h, 3–4·5 h, or 4·5–6 h of stroke. We tested whether the response to alteplase differed by the extent of early ischaemic signs (IST-3 score and ASPECTS score 0–7 *vs* 8–10), and we did a prespecified meta-analysis of data from IST-3 with imaging data from other thrombolysis trials. We judged p values less than 0·05 significant, except for analyses of interactions between imaging signs and response to alteplase, for which we used a significance level less than 0·01, to minimise false-positive results.

This trial is registered at ISRCTN.com, number ISRCTN25765518.

### Role of the funding sources

The funding sources had no role in study design, data collection, data analysis, data interpretation, or writing of the report. All authors had full access to all data in the study and the corresponding author had final responsibility for the decision to submit for publication.

## Results

**Figure 1:**
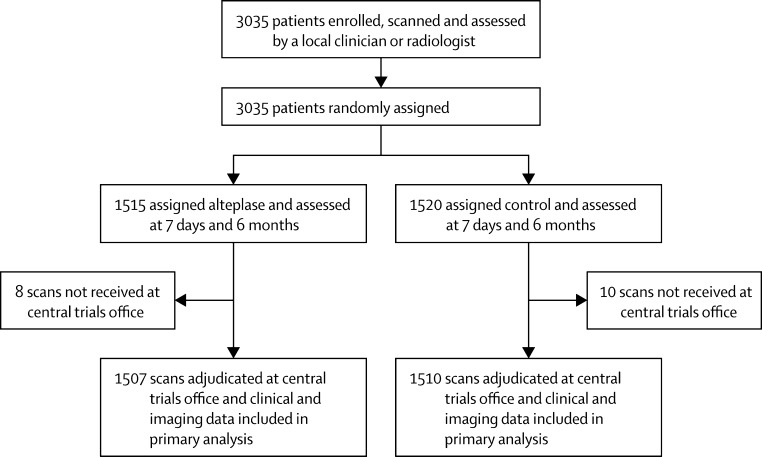
Trial profile

Between May 1, 2000, and July 31, 2011, 3035 patients were recruited to IST-3 and underwent randomisation. Brain scans were available for 1507 patients assigned to alteplase and for 1510 controls; scans for 18 patients were not received at the central trials office and, therefore, were excluded from analyses (figure 1). Table 1 shows baseline clinical and imaging characteristics, which were well balanced between arms and no variables were missing.

Of 3017 prerandomisation brain scans, 2962 (98%) were obtained by CT and 55 (2%) by MRI. The expert panel judged 269 (9%) scans normal (ie, no early ischaemic or pre-existing signs); 1224 (41%) patients had early ischaemic signs (ie, visible infarct, whether or not pre-existing signs were also present) and 1524 (51%) had pre-existing signs (but no early ischaemic signs). The commonest early ischaemic sign was tissue hypoattenuation, seen in 1203 (40%) patients, and the least frequent sign was hyperattenuated artery, recorded in 735 (24%) patients (table 1). Pre-existing signs were common: 1336 (44%) patients had signs of an old infarct, 1547 (51%) had leukoaraiosis, and 2327 (77%) had evidence of atrophy.

Some strong associations were noted between individual imaging variables and age, NIHSS score, and time to randomisation (table 2; appendix pp 20–22). Every point increase in NIHSS score was associated with a roughly 10% increase in the odds of tissue hypoattenuation, swelling, hyperattenuated artery, or large lesion. Every delay of 1 h increased the odds of tissue hypoattenuation, but not other early or pre-existing structural signs. Every increase in age by 1 year decreased the odds of tissue hypoattenuation, large lesion, swelling, or hyperattenuated artery by about 2% (table 2), but the odds of old infarct, atrophy, and leukoaraiosis were increased.

Individually, all early ischaemic signs predicted worse outcomes (table 3) with the exception of severe hypoattenuation, although relatively few patients had this sign. Individually, of all pre-existing signs, only old infarct predicted symptomatic haemorrhage (table 2); no pre-existing signs predicted death within 7 days. Leukoaraiosis and severe atrophy predicted death by 6 months, all pre-existing structural signs predicted reduced chance of being alive and independent (OHS score of 0–2) at 6 months, and leukoaraiosis and atrophy predicted diminished chance of a favourable outcome (OHS score of 0–1) at 6 months. Similar associations between imaging signs and outcomes were seen for patients randomised within 3 h, 3–4·5 h after stroke, and 4·5–6 h after stroke (appendix pp 6–7).

The multivariate logistic regression model for symptomatic intracranial haemorrhage included age, NIHSS score, time to randomisation, individual imaging signs, alteplase, and use of antiplatelet drugs immediately before stroke.^12^ Increasing NIHSS score, alteplase, antiplatelet treatment, and pre-existing old infarcts predicted a significantly higher risk of symptomatic intracranial haemorrhage (table 4). With standard stepwise model selection methods, retaining age, NIHSS score, time to randomisation, and treatment group, both old infarcts and hyperattenuated arteries were potentially significant predictors of symptomatic intracranial haemorrhage (table 5). The multivariate logistic regression model for good functional outcome (OHS score of 0–2) at 6 months showed that increasing age and NIHSS score were the strongest adverse predictors, but hyperattenuated arteries, large lesion, and leukoaraiosis each individually predicted less chance of a good outcome by about 25–30% (table 4). With standard stepwise regression methods, the selected model retained large lesion, hyperattenuated arteries, and leukoaraiosis, with coefficients very similar to those in the full model (table 5). Similar effects were seen in patients randomised 0–3 h, 3–4·5 h, and 4·5–6 h after stroke (appendix pp 8–9). However, potential differences—eg, that tissue hypoattenuation (rather than old infarcts or hyperattenuated arteries) predicts symptomatic intracranial haemorrhage in patients randomised 4·5–6 h after stroke—should be interpreted with caution because of the weak significance level and smaller sample.

**Figure 2:**
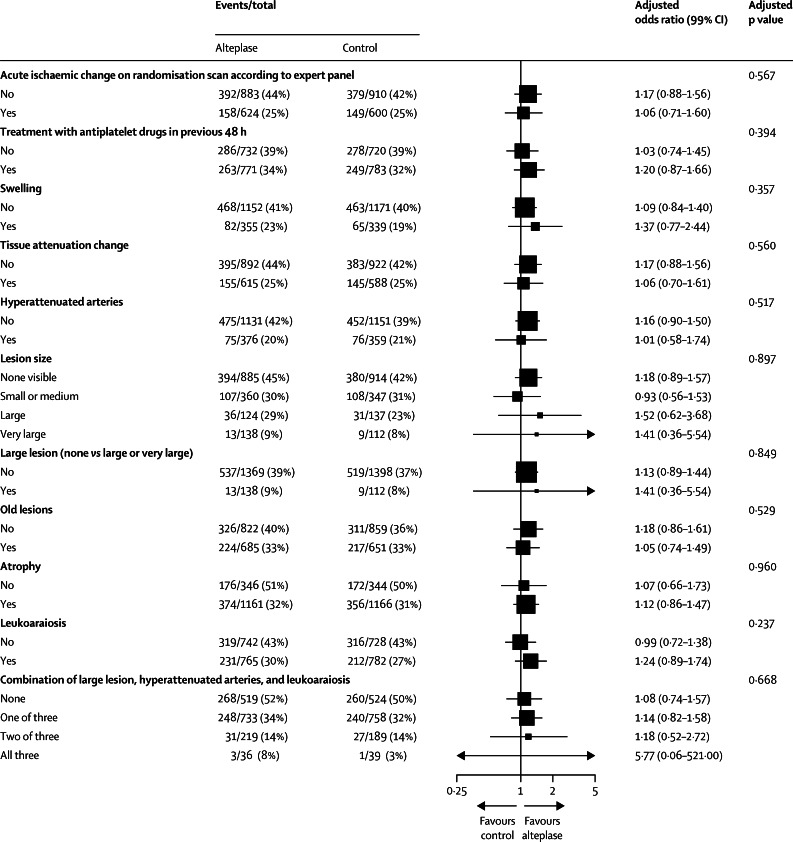
Forest plot showing the adjusted effect of treatment and baseline imaging signs on Oxford Handicap Scale score 0–2 at 6 months Data are adjusted for age, National Institutes of Health Stroke Scale score, and time to randomisation.

**Figure 3:**
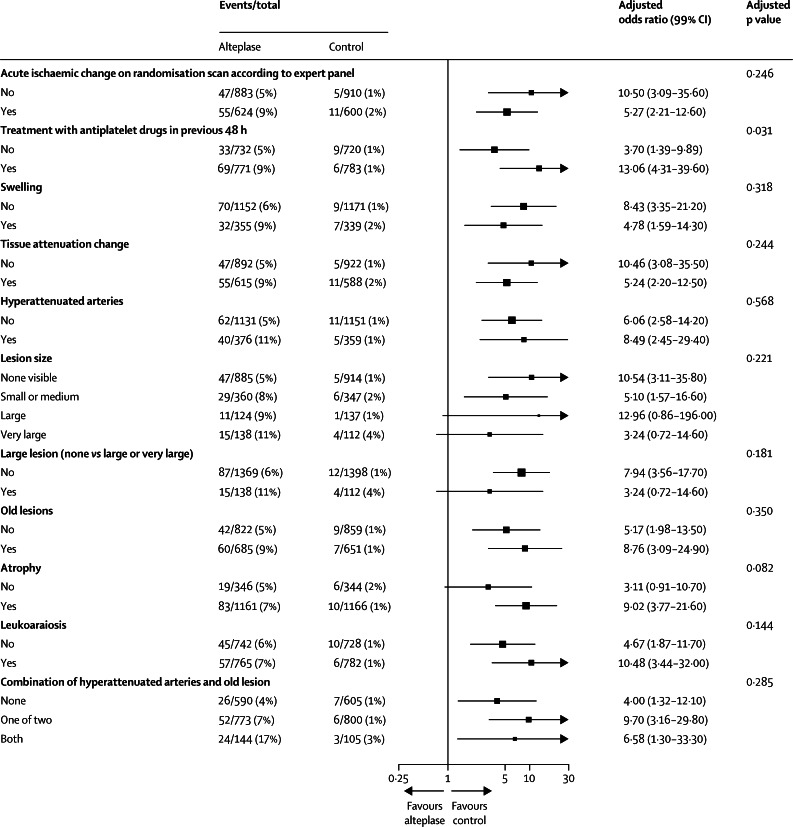
Forest plot showing the adjusted effect of treatment and baseline imaging signs on symptomatic intracranial haemorrhage within 7 days Data are adjusted for age, National Institutes of Health Stroke Scale score, and time to randomisation.

No interaction was recorded between any individual or combined imaging variable and alteplase, for either functional outcome (OHS score of 0–2, figure 2) or symptomatic intracranial haemorrhage (figure 3). Furthermore, no interaction was noted between any individual or combined image variable and alteplase in the ordinal shift analysis or in analyses restricted to patients randomised within 3 h of stroke or 3–4·5 h or 4·5–6 h after stroke (appendix pp 10–17).

Despite the absence of definite interactions between imaging signs and alteplase, the absolute increase in symptomatic intracranial haemorrhage after alteplase with combined imaging signs was substantial. The combination of old infarcts and hyperattenuated arteries (adjusting for age, NIHSS score, and time to randomisation) predicted nearly three-fold increased odds of symptomatic intracranial haemorrhage (odds ratio 2·98, 95% CI 1·71–5·16) versus patients with neither sign (both signs present, absolute excess of events with alteplase 13·8%, 95% CI 6·9–20·7; both signs absent, absolute excess with alteplase 3·2%, 1·4–5·1; appendix p 18). Similar absolute effects on symptomatic intracranial haemorrhage were seen for patients randomised within 3 h of stroke (appendix pp 12–14). A difference in absolute effects was not seen for functional outcome at 6 months (OHS score of 0–2) in the whole study group (appendix p 19) and by separate time windows (data not shown).

## Discussion

**Figure 4:**
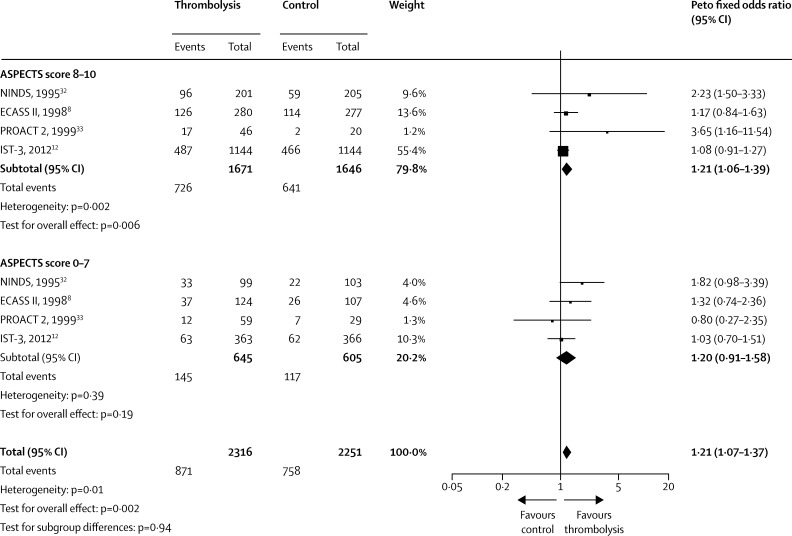
Forest plot showing the interaction between response to alteplase and early infarct size in previous trials ASPECTS=Alberta Stroke Programme Early CT Signs. ECASS II=European Co-operative Acute Stroke Study-II. IST-3=third International Stroke Trial. NINDS=National Institute of Neurological Disorders and Stroke. PROACT 2=Prolyse in Acute Cerebral Thromboembolism 2.

To date, IST-3 is the largest randomised controlled trial of thrombolysis versus control after ischaemic stroke in patients for whom alteplase was judged promising but unproven. Although IST-3 was neutral on the primary endpoint (OHS score of 0–2),^12^ this finding does not preclude the presence of clinically relevant interactions with treatment. Therefore, we planned a priori a detailed secondary analysis of the association of imaging signs with thrombolysis effects. Imaging signs are powerful prognostic markers; however, we recorded no unequivocal evidence that any individual imaging sign modified response to alteplase in patients presenting up to 6 h after ischaemic stroke. In this population, no interaction was noted between extensive early ischaemia—commonly cited as an exclusion for thrombolysis treatment—and alteplase, which accords with previous findings (panel, figure 4).^6,8,12,22,31–38^ Some combinations of imaging features (eg, old infarct and hyperattenuated artery) were associated with an increased absolute excess of symptomatic intracranial haemorrhage after alteplase.

The plain CT scans obtained in IST-3 reflect imaging done in practice, with speed and accessibility being important factors in view of the steep time dependency of alteplase benefit.^1,2,12,39^ The scans reflected common problems, such as movement of the patient or oblique positioning. To date, the brain imaging dataset in IST-3 is the largest in an acute stroke trial (more than 7000 scans) to be read centrally. Scan reading was structured and masked, and follow-up was complete. We pretested the imaging rating system thoroughly^17,18^ and the assessors were skilled in acute stroke imaging. Although the expert panel might have detected subtle changes in tissue attenuation, we showed previously that those who were less experienced could still identify hyperattenuated arteries, established tissue ischaemia, and pre-existing structural changes with good reliability.^17^ These results should inform clinical practice.

Although more patients were randomised within 3 h of stroke in IST-3 (n=846) than in any other previous trial,^32^ we might have missed an interaction between an imaging sign and alteplase in the subgroup of patients treated within 3 h, because very few symptomatic intracranial haemorrhage events arose in controls, and the estimate of effect on OHS score had wide CIs in each subgroup. Associations between clinical and imaging signs reflect the specific IST-3 population; inference to the wider population with ischaemic stroke—including individuals not judged eligible for the trial—remains speculative. Few patients with obvious tissue hypoattenuation (ie, in whom the acute ischaemic tissue was of lower attenuation than normal grey and white matter) were included in the trial; severe tissue hypoattenuation, including appearances suggesting a lesion older than 6 h, was an exclusion criterion. Additionally, patients with extensive ischaemic lesions (ASPECTS 0–7) comprised only a quarter of the study population, although this proportion is higher than in any previous trial. Hence, although IST-3 was larger than previous trials, it might not have had enough statistical power to ascertain whether alteplase treatment in this subgroup of patients adds risk or benefit. We might have identified a spurious association, but we have been cautious in the interpretation, including 99% CIs for tests of interaction. The use of stepwise methods to identify parsimonious predictive models should not be interpreted as ruling out effects of those imaging signs that were excluded from the models. Different subsets of the full set of predictors could produce almost equally good results in this dataset; therefore, further research to assess the contribution of different signs to the risk of adverse outcomes would be valuable.

Previous analyses of imaging predictors of the response to alteplase focused on individual acute ischaemic signs.^20,40^ Here, we have shown that pre-existing signs are important. The association between symptomatic intracranial haemorrhage and old infarcts could account for why the risk of symptomatic intracranial haemorrhage does not change with increasing time to alteplase up to 6 h;^1,2^ another factor present before the stroke, or that is independent of time after stroke, might increase risk. Worsening of functional outcome with old infarcts, atrophy, and leukoaraiosis might indicate increased susceptibility to acute ischaemia or alteplase hazards and could provide useful markers of so-called brain frailty.^41^ Further research is warranted to ascertain the strength of association between pre-existing signs on brain CT and clinical markers of brain frailty. Our findings also emphasise the value to future trials of minimising easy-to-detect imaging prognostic signs to avoid baseline imbalances.

The presence of combinations of imaging signs in patients after stroke might provide additional information to decision making when clinical uncertainty exists about the likely benefit of alteplase—eg, in a patient presenting close to the latest time window or for whom the likelihood of benefit was marginal.^2^ Additional factors affecting risk of symptomatic intracranial haemorrhage (eg, taking antiplatelet drugs) might contribute to decision making in such patients. A hint, in stepwise modelling, that early ischaemic tissue hypoattenuation might be associated with increased risk of symptomatic intracranial haemorrhage with alteplase 4·5–6 h after stroke (appendix pp 8–9) should be treated with caution, because this finding was not confirmed in the formal test of interaction (appendix pp 12–17). Helpfully for routine practice, the key prognostic CT imaging variables identified here are easy to detect: hyperattenuated artery has the best observer reliability of all early ischaemic signs^20^ across a wide range of observers,^17^ and old infarcts, atrophy, and leukoaraiosis^26,42^ are also easy to detect.^17^ Perceived difficulties in detecting early tissue hypoattenuation might have reduced confidence in use of CT scanning before alteplase.^3^ However, our findings confirm that neither early tissue hypoattenuation nor large infarct extent on ASPECTS score should exclude patients from alteplase, hopefully improving confidence in use of CT scanning in acute stroke.

Correspondence to: Prof J M Wardlaw, Brain Research Imaging Centre, Centre for Clinical Brain Sciences, University of Edinburgh, Western General Hospital, Edinburgh EH4 2XU, UK **joanna.wardlaw@ed.ac.uk**

## Figures and Tables

**Table 1:** Baseline clinical and imaging variables

	**Alteplase (n=1507)**	**Control (n=1510)**
**Age (years)**		
≤80	693 (46%)	715 (47%)
>80	814 (54%)	795 (53%)
18–50	58 (4%)	68 (5%)
51–60	98 (7%)	102 (7%)
61–70	187 (12%)	175 (12%)
71–80	350 (23%)	370 (25%)
81–90	703 (47%)	697 (46%)
>90	111 (7%)	98 (6%)
NIHSS score*		
0–5	303 (20%)	304 (20%)
6–10	419 (28%)	428 (28%)
11–15	304 (20%)	295 (20%)
16–20	268 (18%)	271 (18%)
>20	213 (14%)	212 (14%)
**Time to randomisation (h)**		
0 to ≤3	431 (29%)	415 (27%)
>3 to ≤4·5	575 (38%)	596 (39%)
>4·5 to ≤6	501 (33%)	497 (33%)
>6	0	2 (<1%)
**Randomising clinician's assessment of acute ischaemic change on prerandomisation imaging**		
No change	890 (59%)	892 (59%)
Possibly change	359 (24%)	339 (22%)
Definitely change	258 (17%)	279 (18%)
**Expert reader's assessment of acute ischaemic change on prerandomisation imaging**		
Scan completely normal	140 (9%)	129 (9%)
Scan not normal but no sign of any early ischaemic change	743 (49%)	781 (52%)
Signs of any early ischaemic change	624 (41%)	600 (40%)
**Early ischaemic lesion territory**		
Indeterminate	885 (59%)	914 (61%)
MCA or ACA or border zone	589 (39%)	555 (37%)
Posterior	22 (1%)	36 (2%)
Lacunar	11 (1%)	5 (<1%)
**Early ischaemic lesion changes in MCA territory**		
None	925 (61%)	960 (64%)
One-third or less	357 (24%)	354 (23%)
More than one-third	225 (15%)	196 (13%)
**Early ischaemic lesion size**†		
None visible	885 (59%)	914 (61%)
Small	110 (7%)	97 (6%)
Medium	250 (17%)	250 (17%)
Large	124 (8%)	137 (9%)
Very large	138 (9%)	112 (7%)
**ASPECTS score**‡		
0–4	162 (11%)	138 (9%)
5–7	201 (13%)	228 (15%)
8–10	1144 (76%)	1144 (76%)
**Early ischaemic lesion depth of tissue hypoattenuation**§		
None	892 (59%)	922 (61%)
Mild	503 (33%)	492 (33%)
Severe	112 (7%)	96 (6%)
**Early ischaemic lesion degree of swelling**		
None	1152 (76%)	1171 (78%)
Mild sulcal effacement	283 (19%)	265 (18%)
Mild ventricular effacement	71 (5%)	73 (5%)
Moderate effacement	1 (<1%)	0
Severe effacement	0	1 (<1%)
**Location of hyperattenuated arteries**		
None	1131 (75%)	1151 (76%)
Anterior circulation	360 (24%)	342 (23%)
Posterior circulation	16 (1%)	17 (1%)
ICA, or BA, or MCA and ACA	42 (3%)	33 (2%)
MCA, or ACA, or PCA main	334 (22%)	326 (22%)
**Pre-existing brain changes**		
Evidence of atrophy	1161 (77%)	1166 (77%)
Evidence of leukoaraiosis	765 (51%)	782 (52%)
Evidence of old infarcts	685 (45%)	651 (43%)
Evidence of non-stroke lesions	73 (5%)	77 (5%)

Data are number of patients (%). ACA=anterior cerebral artery. ASPECTS=Alberta Stroke Program Early CT Stroke. BA=basilar artery. ICA=internal carotid artery. IST-3=third International Stroke Trial. MCA=middle cerebral artery. NIHSS=National Institutes of Health Stroke Scale. PCA=posterior cerebral artery.

* Assesses neurological deficit in stroke.

† Refers to IST-3 image reading categorisation of lesion extent (all vascular territories).

‡ Assesses the extent of infarct affecting the MCA territory by subtracting a point for each of ten regions that are involved in the acute ischaemic lesion.

§ Classed as mild (ie, grey matter attenuation had become the same as normal white matter) or severe (ie, grey and white matter attenuation less than normal white matter).

**Table 2:** Logistic linear regression analysis of associations between imaging signs and age, NIHSS score, and time to randomisation

	**Age, adjusted for NIHSS score**		**NIHSS score, adjusted for age**		**Delay, adjusted for age and NIHSS score**	
	Odds ratio (95% CI)	p	Odds ratio (95% CI)	p	Odds ratio (95% CI)	p
**Early ischaemic signs**						
Visible infarct	0·98 (0·97–0·98)	<0·0001	1·11 (1·09–1·12)	<0·0001	1·08 (1·01–1·16)	0·019
Hypoattenuation	0·98 (0·97–0·98)	<0·0001	1·10 (1·09–1·12)	<0·0001	1·10 (1·03–1·18)	0·006
Large lesion*	0·98 (0·97–0·99)	<0·0001	1·11 (1·09–1·13)	<0·0001	1·03 (0·94–1·12)	0·561
Swelling	0·98 (0·97–0·99)	<0·0001	1·09 (1·08–1·11)	<0·0001	1·04 (0·96–1·13)	0·302
Hyperattenuated artery	0·98 (0·97–0·98)	<0·0001	1·10 (1·09–1·12)	<0·0001	1·02 (0·94–1·10)	0·664
**Pre-existing signs**						
Atrophy	1·11 (1·10–1·12)	<0·0001	0·99 (0·98–1·00)	0·179	0·98 (0·89–1·07)	0·621
Leukoaraiosis	1·09 (1·08–1·09)	<0·0001	0·99 (0·98–1·00)	0·221	0·99 (0·93–1·06)	0·843
Old infarct	1·03 (1·03–1·04)	<0·0001	0·99 (0·98–1·00)	0·017	0·98 (0·92–1·05)	0·566

Associations for visible infarct (the summary variable) are provided for completeness. Odds ratios and 95% CIs indicate the increased or decreased odds of the imaging sign being present for a 1 point change in NIHSS score, a 1 year change in age, or a 1 h increase in time to randomisation. Each of the eight imaging variables was used separately in two logistic regressions: first on age and NIHSS score (both linear) to give the values in the first two pairs of columns; and second on age, NIHSS score, and time to randomisation (all linear) to give the values in the last pair of columns. IST-3=third International Stroke Trial. NIHSS=National Institutes of Health Stroke Scale.

* Large lesion defined as a combination of large and very large on IST-3 score.

**Table 3:** Logistic linear regression analysis of associations between individual imaging signs and primary and secondary outcomes, adjusted for age, NIHSS score, and time to randomisation

	**Symptomatic intracranial haemorrhage**		**Death at 7 days or before**		**Death at 6 months or before**		**Alive and independent (OHS score 0–2)**		**Favourable outcome (OHS score 0–1)**	
	Odds ratio (95% CI)	p	Odds ratio (95% CI)	p	Odds ratio (95% CI)	p	Odds ratio (95% CI)	p	Odds ratio (95% CI)	p
**Early ischaemic signs**										
Visible infarct	1·48 (1·00–2·19)	0·049	1·64 (1·24–2·16)	0·0004	1·39 (1·15–1·68)	0·0007	0·67 (0·55–0·81)	<0·0001	0·63 (0·51–0·79)	<0·0001
Hypoattenuation*	1·54 (1·04–2·27)	0·032	1·64 (1·25–2·16)	0·0004	1·39 (1·15–1·68)	0·0007	0·66 (0·55–0·81)	<0·0001	0·62 (0·50–0·78)	<0·0001
Severe hypoattenuation†	1·31 (0·67–2·56)	0·432	1·04 (0·63–1·74)	0·872	0·92 (0·63–1·33)	0·649	0·87 (0·60–1·27)	0·482	0·78 (0·51–1·19)	0·246
Large or very large lesion‡	1·32 (0·85–2·05)	0·218	2·22 (1·67–2·96)	<0·0001	2·07 (1·64–2·60)	<0·0001	0·51 (0·38–0·68)	<0·0001	0·40 (0·28–0·58)	<0·0001
Very large lesion§	1·51 (0·89–2·57)	0·131	3·20 (2·29–4·47)	<0·0001	2·28 (1·68–3·11)	<0·0001	0·29 (0·17–0·47)	<0·0001	0·22 (0·10–0·46)	<0·0001
Swelling	1·31 (0·87–1·97)	0·199	1·55 (1·17–2·06)	0·002	1·43 (1·16–1·77)	0·0008	0·59 (0·46–0·75)	<0·0001	0·55 (0·41–0·73)	<0·0001
Hyperattenuated arteries	1·54 (1·03–2·29)	0·034	1·44 (1·09–1·91)	0·009	1·41 (1·15–1·73)	0·001	0·59 (0·47–0·75)	<0·0001	0·63 (0·48–0·83)	0·001
**Pre-existing signs**										
Any leukoaraiosis¶	1·01 (0·68–1·50)	0·967	1·09 (0·82–1·45)	0·536	1·38 (1·14–1·67)	0·001	0·72 (0·59–0·87)	0·0007	0·62 (0·50–0·76)	<0·0001
Severe leukoaraiosis‖	1·15 (0·77–1·70)	0·499	1·17 (0·89– 1·54)	0·267	1·43 (1·18–1·72)	0·0002	0·66 (0·54–0·80)	<0·0001	0·62 (0·50–0·78)	<0·0001
Atrophy**	0·97 (0·58–1·64)	0·917	0·83 (0·57–1·20)	0·315	1·22 (0·93–1·60)	0·149	0·74 (0·59–0·94)	0·013	0·64 (0·50–0·82)	0·0004
Severe atrophy††	1·02 (0·64–1·63)	0·923	0·87 (0·63–1·22)	0·422	1·28 (1·03–1·59)	0·026	0·79 (0·63–1·01)	0·057	0·75 (0·57–0·99)	0·040
Old infarct	1·72 (1·18–2·51)	0·005	0·94 (0·72–1·22)	0·622	1·05 (0·87–1·26)	0·603	0·88 (0·73–1·05)	0·149	0·79 (0·64–0·96)	0·017

Associations for visible infarct (the summary variable) are provided for completeness. The odds ratio is the estimated odds of an outcome happening when the imaging feature is present, divided by the odds of an outcome happening when the imaging feature is absent. NIHSS=National Institutes of Health Stroke Scale. OHS=Oxford Handicap Scale.

* Mild or severe hypoattenuation versus none.

† Severe hypoattenuation versus mild or none.

‡ Large or very large lesion versus no lesion or small or medium lesion.

§ Very large lesion versus no lesion or small, medium, or large lesion.

¶ Mild or severe leukoaraiosis versus none.

‖ Severe leukoaraiosis versus mild or none.

** Moderate or severe atrophy versus none.

†† Severe atrophy versus moderate or none.

**Table 4:** Full multivariate logistic regression models for symptomatic intracranial haemorrhage and functional outcome at 6 months

	**OHS score 0–2 versus OHS score 3–6**		**Symptomatic intracranial haemorrhage**	
	Odds ratio (95% CI)	p	Odds ratio (95% CI)	p
Age (years)	0·96 (0·96–0·97)	<0·0001	1·00 (0·98–1·02)	0·911
NIHSS score	0·83 (0·82–0·85)	<0·0001	1·06 (1·03–1·10)	<0·0001
Time to randomisation (h)	1·04 (0·96–1·13)	0·303	0·98 (0·83–1·16)	0·814
Alteplase versus control	1·13 (0·94–1·35)	0·192	6·65 (3·89–11·35)	<0·0001
Antiplatelets at the time of stroke versus none	..	<0·0001	1·60 (1·07–2·38)	0·021
Large or very large lesion versus small, medium, or no lesion	0·69 (0·49–0·99)	0·043	0·97 (0·55–1·72)	0·919
Swelling	0·79 (0·56–1·11)	0·168	0·95 (0·54–1·69)	0·867
Hyperattenuated artery	0·70 (0·54–0·91)	0·007	1·45 (0·92–2·28)	0·114
Mild tissue hypoattenuation versus none	1·11 (0·52–2·38)	0·783	0·68 (0·17–2·78)	0·588
Severe tissue hypoattenuation versus none	1·40 (0·61–3·21)	0·432	0·71 (0·15–3·26)	0·658
Old infarcts	0·90 (0·74–1·09)	0·278	1·75 (1·17–2·63)	0·007
Mild leukoaraiosis versus none	0·81 (0·65–1·00)	0·051	1·01 (0·64–1·59)	0·975
Severe leukoaraiosis versus none	0·64 (0·48–0·85)	0·002	0·92 (0·51–1·66)	0·772
Mild atrophy versus none	0·87 (0·67–1·12)	0·273	0·83 (0·47–1·48)	0·532
Severe atrophy versus none	0·72 (0·52–1·00)	0·052	0·82 (0·40–1·67)	0·590

Numbers of patients in each category are shown in table 1. Odds ratios indicate the increased or decreased odds of the outcome being present for a 1 year increase in age, a 1 point increase in NIHSS score, or a 1 h increase in time to randomisation. Hosmer-Lemeshow tests for lack of fit: OHS outcome, p=0·59; symptomatic intracranial haemorrhage outcome, p=0·76. Additional time windows are shown in the appendix (pp 8–9). NIHSS=National Institutes of Health Stroke Scale. OHS=Oxford Handicap Scale.

**Table 5:** Multivariate logistic regression models selected by stepwise logistic regression for symptomatic intracranial haemorrhage and functional outcome at 6 months

	**OHS score 0–2 versus OHS score 3–6**		**Symptomatic intracranial haemorrhage**	
	Odds ratio (95% CI)	p	Odds ratio (95% CI)	p
Age (years)	0·96 (0·95–0·97)	<0·0001	0·99 (0·98–1·01)	0·556
NIHSS score	0·83 (0·82–0·85)	<0·0001	1·07 (1·04–1·10)	0·0001
Time to randomisation (h)	1·04 (0·96–1·13)	0·288	0·98 (0·83–1·15)	0·798
Alteplase versus control	1·12 (0·94–1·34)	0·196	6·71 (3·93–11·46)	<0·0001
Antiplatelets at the time of stroke versus none	..	..	1·60 (1·07–2·38)	0·021
Small or medium lesion versus no lesion	0·85 (0·67–1·07)	0·173	..	..
Large or very large lesion versus no lesion	0·54 (0·39–0·74)	0·0001	..	..
Hyperattenuated artery versus none	0·71 (0·55–0·92)	0·009	1·61 (1·07–2·42)	0·023
Old infarct versus none	..	..	1·67 (1·13–2·46)	0·009
Mild leukoaraiosis versus none	0·76 (0·62–0·93)	0·009	..	..
Severe leukoaraiosis versus none	0·59 (0·45–0·79)	0·0003	..	..

Numbers of patients in each category are shown in table 1. Odds ratios indicate the increased or decreased odds of the clinical factor being present for a 1 year increase in age, a 1 point increase in NIHSS score, or a 1 h increase in time to randomisation. Models selected by stepwise logistic regression from full models shown in table 4. Additional time windows are shown in the appendix (pp 8–9). The first four variables were forced into all models. We used p≤0·05 as criteria for both forward and backward steps. Blank cells represent variables that were dropped as non-significant during stepwise selection. The final nominal p values take no account of model selection. Age, NIHSS score, and time to randomisation were entered into the model as continuous variables; thus the odds ratio for age represents the estimated change in odds of the outcome for a 1 year increase in age, with all other variables unchanged. The units for NIHSS score are points on a scale from 0 to 37 (maximum observed in this trial). Factors with three levels were either retained or excluded at each step; the method did not permit separate consideration of the individual 1 df contrasts comprising the three-level factors. NIHSS=National Institutes of Health Stroke Scale. OHS=Oxford Handicap Scale.

## Panel Research in context

**Systematic review**

We searched the Cochrane Stroke Trials Registry, Medline, and Embase from January, 1990, to March, 2015, to identify all previous randomised controlled trials of thrombolysis versus control in acute ischaemic stroke with data for CT brain imaging and any interaction with thrombolysis.^31^ Three trials provided data for the extent of early ischaemic signs on CT brain imaging and functional outcome, in patients treated by thrombolysis and in controls, dichotomised on the Alberta Stroke Programme Early CT Signs (ASPECTS) score 0–7 and 8–10. Two trials tested intravenous alteplase (0·9 mg/kg)^8,32^ and one tested intra-arterial pro-urokinase.^33^ No other trials had data for imaging signs and outcome by treatment allocation for meta-analysis. In the three previous trials,^8,32,33^ patients with large early ischaemic lesions (ASPECTS score 0–7, n=521) had similar odds of good outcome (odds ratio 1·41, 95% CI 0·95–2·09) to those with small or no lesions (ASPECTS score 8–10, n=1029; odds ratio 1·59, 95% CI 1·24–2·04) after thrombolysis (test for subgroup differences, p=0·61). In the third International Stroke Trial,^12^ patients with large lesions (ASPECTS score 0–7, n=729) had similar odds of good outcome (odds ratio 1·03, 95% CI 0·70–1·51) to those with small or no visible ischaemic lesions (ASPECTS score 8–10, n=2288; odds ratio 1·08, 95% CI 0·91–1·27). Combining all four trials (figure 4) shows that patients with large infarcts (ASPECTS 0–7, n=1250) had similar odds of good outcome (odds ratio 1·20, 95% CI 0·91–1·58) to patients with small or no visible infarct (ASPECTS 8–10, n=3317; odds ratio 1·21, 95% CI 1·06–1·39) after thrombolysis (test for subgroup difference, p=0·94). These findings accord with those of the Interventional Management of Stroke III trial (no interaction between ASPECTS score and thrombectomy)^34^ and trials in which the classification of early ischaemic signs on CT precluded meta-analysis with our data (no interaction between early ischaemic signs and streptokinase in 1292 patients within 6 h,^35^ or alteplase in 624 patients in the National Institute of Neurological Disorders and Stroke trial within 3 h of stroke)^6^ on functional outcome. No data were available in these trials for analysis of pre-existing CT signs and effects of alteplase, nor to adjust for clinical prognostic variables.

**Interpretation**

Absence of an interaction between early ischaemic signs and thrombolysis has been disregarded by many up until now, perhaps because data came (in part) from trials that were neutral or that used a drug perceived to be harmful (eg, streptokinase). All data combined show that the extent of early ischaemic signs on CT per se should not deter treatment with alteplase. A meta-analysis of CT findings from individual patients in all trials of alteplase is planned as part of the Stroke Thrombolysis Trialists' Collaboration, to test further the effect of early ischaemic and pre-existing structural signs and combinations of imaging signs for interactions with alteplase, adjusted for baseline prognosis.^2^ Future trials should not only assess effects of pre-existing and early ischaemic changes for interactions with acute stroke treatments but also consider use of imaging variables for minimisation in view of their strong independent prognostic value.
