# Supplementary material for: Association between brain imaging signs, early and late outcomes, and response to intravenous alteplase after acute ischaemic stroke in the third International Stroke Trial (IST-3): secondary analysis of a randomised controlled trial
Source: Lancet Neurol. 2015 May;14(5):485–96. doi: 10.1016/S1474-4422(15)00012-5 (PMC4513190; doi:10.1016/S1474-4422(15)00012-5)
Supplement: Supplementary appendix [file mmc1.pdf]

THE LANCET Neurology

Supplementary webappendix

This webappendix formed part of the original submission and has been peer reviewed. We post it as supplied by the authors.

Supplement to: The IST-3 collaborative group. Association between brain imaging signs, early and late outcomes, and response to intravenous alteplase after acute ischaemic stroke in the third International Stroke Trial (IST-3): secondary analysis of a randomised controlled trial. *Lancet Neurol* 2015; published online March 27. http://dx.doi.org/10.1016/S1474-4422(15)00012-5.

CT brain imaging, prognosis, and response to intravenous alteplase after acute ischaemic stroke in the Third International Stroke Trial: a secondary analysis of a randomised controlled trial.

Supplementary material

Appendix. IST-3 Committees and Centres

Supplementary Table 1. Association between imaging variables and outcomes, adjusted for age, NIHSS and time to randomisation: a) 0-3 hours, b) 3-4.5 hours, c) 4.5-6 hours.

Supplementary Table 2. Comparison of multiple regression models for SICH and OHS 0-2 in three time windows. A) SICH, B) OHS at six months, i) full models, ii) stepwise logistic regression models.

Supplementary Table 3. Unadjusted and adjusted subgroup effects on ordinal outcome (5 levels: 0, 1, 2, 3, 4-6) at six months.

Supplementary Table 4. Adjusted effect of treatment with alteplase on SICH, Odds Ratio and 99% Confidence Interval, randomisation: A) 0-3 hours, B) 3-4.5 hours, C) 4.5-6 hours; and on OHS 0-2, randomisation: D) <3 hours, E) 3-4.5 hours, and F) 4.5-6 hours.

Supplementary Table 5. **A**. Effect of combinations of imaging signs on SICH: Logistic regression model for SICH with age, NIHSS, time to randomisation, treatment and combinations of old lesions and hyperattenuated artery as predictors. There were 118/3017 patients with SICH. **B**. Absolute excess of SICH events after alteplase as a function of old lesions and hyperdense artery.

Supplementary Table 6A. Effect of combinations of imaging signs on functional outcome: Logistic regression model for odds of six month independent survival (OHS 0-2) with age, NIHSS, time to randomisation, treatment and combinations of large lesions, leukoaraiosis and hyperattenuated artery as predictors. **B**. Absolute excess primary outcome (OHS 0-2) events after alteplase as a function of large lesion, hyperattenuated artery and leukoaraiosis

Supplementary Figure 1 Associations between early ischemic or pre-existing signs on baseline imaging and a) increasing age (all P<0.02 except large lesion; increasing atrophy, leukoaraiosis and old infarcts, all P<0.0001), b) increasing NIHSS (all p<0.0001), and c) increasing time to randomisation (no association with early ischemic signs; pre-stroke signs all P<0.0001). See Table 2 main paper for adjusted Odds ratios.

Appendix: Committees and Participating Centres of the IST-3 collaborative group

National Co-ordinators and Associate Co-ordinators
Australia: Richard Lindley, Graeme J. Hankey. Austria: Karl Matz, Michael Brainin. Belgium: Andre Peeters. Canada: Gord Gubitz, Stephen Phillips. Italy: Stefano Ricci. Mexico: Antonio Arauz. Norway: Eivind Berge, Karsten Bruins Slot. Poland: Anna Czlonkowska, Adam Kobayashi. Portugal: Manuel Correia. Switzerland: Phillippe Lyrer, Stefan Engelter. Sweden: Veronica Murray, Bo Norrving, Andreas Terént, Per Wester: UK: Graham Venables.

Trial Steering Committee:
Independent Chairman, Professor Colin Baigent (University of Oxford), Professor David Chadwick (Former Chairman, Retired)): University of Liverpool; Independent members: Dr Pippa Tyrrell (Manchester University), Professor Gordon Lowe (Glasgow University); Co-principal Investigators: Professor Peter Sandercock (University of Edinburgh); Professor Richard Lindley (Sydney Medical School – Westmead Hospital, and the George Institute for Global Health, University of Sydney); Chief Investigator for Neuroradiology: Professor Joanna Wardlaw (University of Edinburgh); Professor Martin Dennis (University of Edinburgh); Statistician: Geoff Cohen; Trial Co-ordinator: Karen Innes. Lay representative: Heather Goodare.

Data Monitoring Committee
Professor Rory Collins, Oxford University, UK (Chairman), Professor Philip Bath (Nottingham University), Professor Jan van Gijn (former member, now retired) (University of Utrecht, The Netherlands), Professor Richard Gray (University of Oxford), Professor Robert Hart (McMaster University, Canada), Professor Salim Yusuf (McMaster University, Canada).

Event Adjudication Committee
Keith Muir, Peter Sandercock, Richard Lindley.

CT and MR reading panel
Joanna Wardlaw, Andrew Farrall, Rüdiger von Kummer, Lesley Cala, Anders von Heijne, Zoe Morris, Alessandro Adami, Andre Peeters, Gillian Potter, Nick Bradey.

Trial Coordinating Centre, Edinburgh. Karen Innes, Alison Clark, David Perry, Vera Soosay, David Buchanan, Sheila Grant, Eleni Sakka, Jonathan Drever, Pauli Walker, Indee Herath, Ann Leigh Brown, Paul Chmielnik, Christopher Armit, Andrea Walton, Mischa Hautvast, Steff Lewis, Graeme Heron, Sylvia Odusanya, Pam Linksted, Ingrid Kane, Will Whiteley, Robin Sellar, Philip White, Peter Keston, Andrew Farrell, Zoe Morris, Hector Miranda. **CTSU, Oxford**, Lisa Blackwell. **National Co-ordinating centres**: ***Italy*** up to September 2008: Maria Grazia Celani; Enrico Righetti. After September 2008: Silvia Cenciarelli; Tatiana Mazzoli. Central Follow up for Italy: Teresa Anna Cantisani. ***Poland*** Jan Bembenek. ***Sweden*** Eva Isaksson. ***Norway*** Eivind Berge, Karsten Bruins Slot.

List of members of IST-3 collaborative group and participating hospitals in each country.
Figures in parentheses are the number of patients recruited in the country or by the centre. ***UK*** *(1447)* Royal Hallamshire Hospital (118): G Venables, C Blank, H Bowler, C Doyle, K Endean, K Harkness, E Parker, M Randall. University Hospital of North Staffordshire (97): C Roffe, N Ahmad, A Arora, S Brammer, J Chembala, B Davies, S Ellis, E Epstein, K Finney, C Jackson, C Jadun, R Kinston, H Maguire, I Memon, I Natarajan, M Poulson, R Sanyal, S Sills, A Vreeburg, E Ward. Western General Hospital (95): P Sandercock, R Al-Shahi Salman, R Davenport, M Dennis, P Hand, S Hart, I Kane, S Keir, M MacLeod, L McKinlay, H Milligan, E Sandeman, J Stone, C Sudlow, P Taylor, J Wardlaw, C Warlow, W Whiteley, A Williams. The National Hospital for Neurology & Neurosurgery (84): M Brown, B Athwal, V Bassan, N Bhupathiraju, J Bowler, C Davie, D Doig, R Erande, S Gilbert, L Ginsberg, R Greenwood, S Gregoire, N Harding, N Losseff, R Luder, N Passeron, R Perry, P Rayson, R Simister, S Stone, D Werring. Arrowe Park Hospital (83): J Barrett, H Aitken, S Cherian, R Davis, S Downham, L Godd, V Gott, D Jose, V Little, D Lowe, L Luxford, M McGrory, P Owings, N Price, J Richards, G Sangster, J Sherlock, S Vargese, I Wakefield, P Weir. Southend University Hospital (77): P Guyler, T Attygale, S Chandler, L Coward, S Feasey, C Khuoge, T Loganathan, S Martin, A O'Brien, D Sinha, V Thompson, S Tysoe, R Walsh. Norfolk and Norwich University Hospital (67): K Metcalf, J Cochius, R Fulcher, N Gange, C Green, J Jagger, M Lee, P Myint, J Potter, G Ravenhill, S Shields, N Shinh, T Staunton, E Thomas, W Woodward, P Worth, N Wyatt. Nottingham City Hospital (63): W Sunman, P Bath, P Berman, J Clarke, C Gaynor, F Hammonds, R Harwood, K Mitchell, S Munshi, S Pacey, A Shetty, N Sprigg, H Stear, G Subramanian, A Wills. Guy's & St.Thomas Hospital (60): A Rudd, H Audebert, A Bhalla, J Birns, R Chowdhury, G Cluckie, I Davies, C Gibbs, P Holmes, N Mitchell, F Schiavone, E White, M Yeung. Darlington and Bishop Auckland Hospitals (56): A Mehrzad, V Baliga, E Brown, L

Burnside, B Esisi, J Kent, P Orr, D Stead, E Wayman. University Hospital Aintree (46): R Durairaj, C Cullen, R Kumar, H Martin, D McDowell, A Sharma, V Sutton, R White. University Hospital of Wales (46): T Hughes, K Ali, J Anderson, K Baker, K Bethune, K Bethune, M Booth, M Cossburn, S Halpin, M Hourihan, E Marsh, K Peall, R Powell, H Shetty, M Wardle, M Williams. Derby Royal Hospital (37): K Muhiddin, J Beavan, M Clarke, R Donneley, S Elliott, P Fox, P Gorman, M Harper, M Mangoyana, I Memon, L Mills, L Wright. Addenbrookes Hospital (34): L Warburton, J Baron, P Barry, D Day, T Harold, P Martin, J Mitchell, E O'Brien, J Rycarte, M Turnham. St George's Healthcare NHS Trust (34): G Cloud, L Choy, B Clarke, C Griffin, O Halse, I Jones, F Kennedy, U Khan, R Lewis, A Loosemore, C Lovelock, H Markus, B Moynihan, J O'Reilly, O Paul, A Pereira, M Punter, P Rich, D Rolfe, F Schiavone. Royal Devon & Exeter Hospital (Wonford) (30): M James, J Bell, A Bowring, L Boxall, J Cageao, H Eastwood, S Elyas, F Hall, S Harries, A Hemsley, S Jackson, S Keenan, P Mudd, A Sekhar, D Strain, J Sword, N Wedge. Aberdeen Royal Infirmary (26): M MacLeod, M Bruce, A Joyson, M Kemp, K McMullan, J Reid, O Robb, J Webster, S Wilkinson. Hammersmith Hospitals & Imperial College (24): P Sharma, P Bentley, H Jenkins, A Kar, T Sachs. Northwick Park Hospital (20): D Cohen, R Bathula, J Devine, M Mpelembue. William Harvey Hospital (20): D Hargroves, I Balogun, L Cowie, A Maidment, D Rand, J Rowe, H Rudenko, D Smithard, L Wray. Scarborough Hospital (17): J Paterson, J Brown, J Hampton, S Jamieson, R Rose, A Volans. Countess of Chester Hospital NHS Foundation Trust (17): K Chatterjee, G Abbott, R Brookes, C Castle, C Kelly, S Leason, A Nallasivan, A Sen. Watford General Hospital (17): D Collas, M Cottle, N Damani, P Jacob, D Oza, D Werring. University Hospitals Coventry & Warwickshire NHS Trust (15): A Kenton, N Adab, L Aldridge, H Allroggen, Y Brown, R Cross, L Galvin, K Ghosh, A Grubneac, A Lindahl, H Mehta, M Pritchard, C Randall, P Ray, A Shehu, S Thelwell. Royal Bournemouth & Christchurch NHS Trust (12): D Jenkinson, J Bell, T Black, O David, J Kwan, A Orpen, C Ovington, D Tiwari, Z ud Din Babar. Leeds General Infirmary (12): A Hassan, A Bailey, J Bamford, C Bedford, R Bellfield, J Cooper, L Dunsmure, J Greig, M Keeling, L Mandizvidza, J Rankine, E Roberts, P Wanklyn, T Webb, S Williamson. York Health Services NHS Trust (12): J Coyle, S Crane, C Croser, P Duffey, R Evans, E Iveson, M Keeling, G Kitching, M Porte, C Rhymes. Queen Elizabeth Hospital (Gateshead) (12): D Barer, M Armstrong, M Bokhari, T Cassidy, B McClelland. Queen Elizabeth The Queen Mother Hospital (10): G Gunathilagan, P DOLKE, S Jain, S Jones, A Maidment, L Rosser, G Thomas, C White. Worcestershire Royal Hospital (10): P Sanmuganathan, C Scholtz, E Stratford. Blackpool Victoria Hospital (10): M O'Donnell, H Goddard, G Hoadley, J Howard, S Leach, J McIlmoyle, A Stewart, A Strain. Basildon & Thurrock University Hospitals NHS FT (9): F Huwez, P Croot, N Gadi, N Mguni, U Umasankar. Royal Infirmary of Edinburgh (8): G Mead, B Chapman, A Coull, S Hart, A Kinnear, B Morrow, F Morrow. St Mary's Hospital (8): D Ames, J Ball, S Bannerjee, J Chataway. Yeovil District Hospital (8): K Rashed, C Buckley, D Donaldson, D Hayward, C Lawson. Luton and Dunstable Hospital (8): L Sekaran, K Bharaj, F Justin, G Jutlla, D Phiri, S Sethuraman, M Tate. Solihull Hospital, Heart of England NHS Trust (8): D Sandler, P Carr, G Jones, J Lyons, K Warren. King's College Hospital (7): L Kalra, A Davis, J Jarosz, D Manawadu, L Sztriha. Doncaster Royal Infirmary (7): D Chadha, A Holford, P Willcoxson. Royal United Hospital Bath (7): L Shaw, D Button, A Cunningham, L Dow, J Dutson, T Hall, C Hardy, N Jakeman, P Kaye, B Madigan, K O'Brien, D Pressdee, M Price, L Robinson, C Taylor, D Williamson. Birmingham Heartlands Hospital (6): D Sandler, P Carr, J Lyons, J McCormack, C Stretton. University Hospital North Durham (6): P Earnshaw, E Brown, S Bruce, C Church, S Desai, B Esisi, M Myint, N Watt. Wansbeck General Hospital (6): C Price, S Elliott, H Graham, R Lakey, K Mitchelson. Bristol Royal Infirmary (6): P Murphy, L Ball, S Caine, J Dovey, J Hughes, A Steele. Stepping Hill Hospital (6): K Dizayee, A Brown, T Chattopadhyay, J Cheetham, H Cochrane, A Datta, M Datta-chaudhuri, C Fox, D Kilroy, S Krishnamoorthy, F Levy, S Metha, P Ngoma, B Venkatesh. Princess Royal Hospital Brighton & Sussex University Hospitals Trust (5): K Ali, R Gautam, N Henderson, M Jones, S Murphy, G Spurling. Belfast City Hospital (5): I Wiggam, C Boyd, K Fullerton, P Gray, M Kinnaird, S MacNair, C Morgan, M Reid, S Tauro. Royal Liverpool University Hospital (5): S Loharuka, D Balmforth, P Cox, G Fletcher, A Ledger, A Manoj, M Wilkinson. City Hospital, Sandwell & West Birmingham Hospital (5): D Nicholl, S Clegg, S Hurdowar, S Kausar, K Law, A Singal, S Sturman. Royal London Hospital (4): P Gompertz, J Evanson, A Farrell, A Petrou, K Saastamoinen, T Sachs, A Salek-Haddadi, R Yadava. Sunderland Royal Hospital (4): J O'Connell, H Brew, S Butler, S Crawford, C Gray, D Gulliver, N Majmudar, R O'Brien. Morriston Hospital (4): M Wani, L Dacey, L Davies, R Evans, D Harris, T Jones, S Storton. Royal Preston Hospital (4): S Punekar, A Ashton, S Duberley, H Emsley, C Gilmour, B Gregary, L Hough, S Philip, S Wuppalapati. The Royal Wolverhampton Hospitals NHS Trust (4): K Fotherby, P Bourke, D D'Costa, K Kauldhar, D Leung, R Lodwick, S McBride, D Morgan, M Qaiyum, G Sahota, M Srinvasan. Royal West Sussex NHS Trust, St Richard's Hospital (4): I Kane, N Chuter, L Garrad, M Hookway, S Ivatts, G Kennedy. Queen's Hospital Romford (4): K Darawil, L Al Dhahirl, S Andole, M Baig, P Dugh, K Dunne, H Kariuki, M Khan, S Rathnayaka. Ulster Hospital (3): M Power, K Dynan, J Finnerty, A Heaney, C Leonard, K McKnight, J Turkington, B Wroath. Great Western Hospital (3): B Dewan, S Cotton, M Gardiner, T Saunders, B Vincent. The Queen Elizabeth Hospital Birmingham (3): D Sims, P Guest, E Jones, J McCormack, D Nicholl, J Savanhu, R Tongue, M Willmot. Leicester General Hospital (3): D Eveson, S Dawson, M Dickens, M Fotherby, R Hunt, S Khan, T Kumar, R Marsh, A Mistri, T Robinson, J Thompson. Darent Valley Hospital,

Dartford & Gravesham NHS Trust (3): P Aghoram, T Daniel, M Gatehouse, S Hussein, A Jackson, T Shanganya, E Strachan, G Tan. Nevill Hall Hospital, Aneurin Bevan Local Health (3): B Richard, S Elaine, S Hanson, S Mosely, H Reed, M Williams. Colchester Hospital University Foundation Trust (3): R Saksena, S Cook, D Demuran, M Keating, R Needle, V Paramsothy, A Sebastian, R Sivakumar, A Wright. Salford Royal Hospital Foundation NHS Trust (2): R Grue, E Barberan, C Dickson, C Douglas, J Jellicoe, T Marsden , J Priestley, E Quick, C Sherrington, A Singh, C Smith, J Stevens, P Tyrell, J Wainwright. Leicester Royal Infirmary (2): M Ardron, J Birchall. Queen Elizabeth Hospital (Kings Lynn) (2): R Shekhar, C Barsted, S Coleman, S Fletcher, J Graham. John Radcliffe Hospital (2): A Buchan, J Hinkle, J Kennedy, A Manoj, M Westwood. Derriford Hospital (2): A Mohd Nor, S Allder, B Hyams, A Pace. West Cumberland Hospital (1): E Orugun, C Brewer, L Huntley, R Jolly, C Summers. Sandwell General Hospital (1): K Sharobeem, J Khaira, J Leahy, E Linehan, G Moore, J Rizkalla, J Wilkinson. Torbay Hospital (1): D Kelly, C Hilaire. Warrington & Halton Hospitals NHS Foundation Trust (1): O Otaiku, L Connell, G Delaney-Sagar, G James, L Lomax, D Matthew, J Simpson, H Whittle. Medway Maritime Hospital (1): S Sanmuganathan, S Burrows, A Mahmood. Southampton General Hospital (1): G Durward, S Barker, J Cantle, P Crawford, S Evans, V Pressly, N Weir. Victoria Hospital (1): V Cvoro, K McCormick. ***Poland*** *(347)* 2nd Department of Neurology, Institute of Psychiatry & Neurology (190): A Czlonkowska, J Bembenek, M Bilik, G Chabik, W Czepiel, J Dzierka, M Gluszkiewicz, K Grabska, B Janus-Laszuk, J Jedrzejewska, A Kobayashi, T Litwin, A Oskedra, A Piorkowska, M Skowronska, A Sliwinska, U Stepien. SPZZOZ w Sandomierzu (43): P Sobolewski, A Gajewska, M Grzesik, R Hatalska-Zerebiec, I Labudzka, B Loch, A Medrykowska, M Sledzinska, A Sobota, W Szczuchniak, G Wolak, I Zdyb. Medical University of Gdansk (35): W Nyka, D Gasecki, K Chwojnicki, A Gojska, B Karaszewski, G Kozera, M Kwarciany, M Nowak, M Swierkocka-Miastkowska , S Szczyrba, M Wisniewska, E Wnorowska. 1st Department of Neurologyt, Institute of Psychiatry & Neurology (25): P Richter, A Bochynska, M Chahwan, A Graban, R Rola. Military Medical Institute (24): A Stepien, B Brodacki, M Grotowska, J Kotowicz, J Staszewski, J Swistak, S Zaloga. Szpital Powiatowy (14): J Stoiñski, K Czajkowaka-Fornal, P Czubak, A Kaczor, J Kraska, E Nowakowska-Sledz, J Ozdoba-Rot, E Zawadzka. Szpital Specjalistyczny w Konskich (8): M Fudala, D Adamczyk, W Brola, I Guldzinska, K Kaluzny, M Kucharska-Lipowska, M Mosiolek, M Polewczyk, M Ziomek. Central University Hospital (7): G Opala, M Arkuszewski, M Kudlacik, P Malgorzata, M Swiat. SPSK im. Prof. W. Orlowskiego CMKP (1): U Fiszer, M Lenska-Mieciek. ***Italy*** *(326)* Ospedale Di Citta' di Castello (62): S Cenciarelli, A Barilaro, R Condurso, F Coppola, S Dioguardi, E Gallinella, A Mattioni, C Menichetti, S Ricci. Nuovo Ospedale Civile "S.Agostino-Estense" (40): F Casoni, M Bacchelli, M Cavazzuti, M Malagoli, A Zini. Ospedale Beato Giacomo Villa - Citta' della Pieve (36): G Benemio, M Celani, R Allegrucci, V Bondo, S Cupella, L Guerra, S Guerrieri, C Ottaviani, E Righetti, C Rossi, N Sacchi, M Scucchi, V Stefanini. Ospedale Di Branca (36): T Mazzoli, A Bigaroni, L Greco, R Paris, P Parise, S Ricci. A.O. Niguarda Ca'Granda (25): A Ciccone, L Basso, R Causarano, P Doneda, E Ferrante, A Gatti, A Guccione, A Gullo, F Imbesi, S Jann, R Marazzi, E Moro, C Motto, D Parodi , A Protti, M Riva, A Rosiello, I Santilli, R Sterzi, P Tiraboschi, G Venturelli. R.Guzzardi Hospital - Vittoria (RG) (19): F Iemolo, R Campagna, G Campagnolo, A Carnemolla, N D'Apico, G D'Asta, S Giannarita, A Giordano, E Sanzar. Ospedale Regionale di Aosta (17): E Bottacchi, S Cordera, G Corso, M Di Giovanni, G Giardini, C Lia, T Meloni, M Pesenti Campagnoni, P Tosi. Ospedale Sacro Cuore - Negrar Verona (12): A Adami, G Rossato, T Zuppini. Ospedale Maggiore - Bologna (12): G Procaccianti, T Sacquegna. Universita degli Studi di Genova (11): C Gandolfo, M Balestrino, C Bruno, L Castellan, M Del Sette, A Ferrari, C Finocchi, N Reale, D Rizzi. Ospedale Civlle S.Andrea (9): M Del Sette, L Benedetti, C Capellini, E Carabelli, E Cibei, M Godani, G Guariglia, E Landini, A Mannironi, B Nucciarone, S Parodi, S Tonelli, E Traverso, D Zito. Ospedale S. Giovanni Battista - Foligno (8): P Brustenghi, F Corea, O Flamini, S Lolli, G Pelliccia, R Ricci, S Stefanucci, M Zampolini. Ospedale a Vibo Valentia (8): D Consoli, F Galati, P Postorino. Azienda Ospedaliero-Universitaria "Ospedali Riuniti" di Foggia (8): G Rinaldi, E Carapelle, G Grilli, M Guido, L Specchio. Ospedale Valduce di Como (8): N Checcarelli, G Borin, L Chiveri, R Clerici, E Corengia, L Gandola, P Garavaglia, M Guidotti, A Martegani, M Mauri, F Muscia, F Raudino. Ospedale di Cattinara - Trieste (4): F Chiodo Grandi, A Bratina, N Carraro, M Gaio, A Granato, N Koscica, M Naccarato, V Sarra, P Schincariol, C Vilotti, Z Zugna. Clinica Dr Pederzoli Spa (4): D Idone, C Bonato, E De Angelis, A Forgione, M Gambera, F Recchia, S Tamburin, P Tinazzi Martini, G Zanette. Ospedale Civile San Matteo Degli Infermi - Spoleto (2): S Grasselli. Ospedale Silvestrini - Perugia (2): G Agnelli, A Andrea, A Billecia, V Caso, V Casso, R Fabiola, P Fanelli, M Paciaroni, B Sergio, M Vemti. Mater Salutis Hospital, Legnago VR (2): M Silvestri, L Altarini, A Bonfante, M Bonornetti, B Costa, N D'Attoma, N Deluca, F Frattini, R Niego, D Rafaele, V Ravenna, M Turazzini. Ospedale Guglielmo da Saliceto - Piacenza (1): S Cammarata. ***Sweden*** *(297)* Uppsala University Hospital (100): E Lundström, L Jonsson , U Söderström, A Terént. Danderyd Hospital (46): V Murray, A Alvelius, M Arbin von, I Dalenbring, Å Doverhall, Å Franzén-Dahlin, N Greilert, M Hallberg, A Heijne von, E Isaksson, H Kumpulainen, A Laska, A Lundström, C Martin, J Muhrbeck, E Näslund, N Ringart, E Rooth, R Undén, P Waldenström. Hassleholm Hospital (29): M Esbjornsson, M Petranek. Karnsjukhuset (25): B Cederin, E Bertholds, A Elgåsen, T Johansson, B Witteborn. Koping Hospital (20): M Kwiatkowska, E Gustafsson, T Noren, J Saaf. Mora Hospital (17): J Teichert, M Bertilsson, S Nilsson, S Oestberg. LidkopingHospital (11): L Welin, K Fredricson, L Pehn. Falu Hospital (11): J

Hambraeus, I Lonn. Capio S: tGoran Hospital (9): B Hojeberg, A Adolfsson, M Anzen. Vastervik Hospital (5): T Wallen, R Schloenzig, P Söderström, A Wennerberg. University Hospital MAS (5): F Buchwald, K Abul-Kasim, A Berkeskold, J Petersson, E Poromaa. University Hospital of Northern Sweden (4): P Wester, R Backlund, A Sjöström. Helsingborgs lasarett (4): B Hedström , E Campbell, K Johnsson, B Karlsson, N Lekokotla, C Lundahl, A Risedal, P Sandgren, A Svensson. Visby Hospital (4): S Bysell, E Smedberg, A Vestberg Bysell. Sundsvall Hospital (3): V Sjögren, B Högvall. University Hospital Lund (2): G Andsberg, T Cronberg, A Lindgren. Vasteras Hospital (1): H Wannberg, F Ax, L Nyren. Karlstad Central Hospital (1): J Sanner, H Andersson, F Andler, S Holmgård, R Johansson, I Magnussan, K Nilsson, J Rådberg. ***Norway*** *(204)* Trondheim University Hospital (69): B Indredavik, H Ellekjær, A Østvik, G Rohweder, D Steckhan, J Storvold. Oslo University Hospital Sykehus (66): E Berge, Y Rønning, R Aakvik, K Bruins Slot, G Knutsen, M Moxness, R Pettersen, T Wyller. University Hospital of North Norway (23): C Wahl, O Iversen, S Johnsen, B Norderhus, L Steffensen, E Stensland. Kongsvinger Hospital (13): T Asak, J Aaseth, T Rotnes, J Sparby, S Wetterhus. Levanger Hospital (9): H Hallan, A Aardal, T Graven, H Hansbakk Skjetne, B Klykken, K Lindqvist, A Tommy. University Hospital of North Norway (8): T Engstad, M Antonsen, R Bajic, W Fønnebø, S Hykkerud, I Lyngmo, A Nyrnes, S Rogne, S Sparr. Harstad Hospital (7): O Kildahl-Andersen, K Pedersen, H Ulrichsen. Ålesund Hospital (4): O Skogen, I Alnes, R Hukari, Y Seljeseth, P Vadset. Asker and Bærum Hospital (2): G Knutsen, B Fure, H Ihle-Hansen, N Johnsen, L Kornberg. Namsos Hospital (2): S Schuler, M Heibert. Volda Hospital (1): M Lillebø, O Aasen, I Eskeland, T Hamre, S Hareide, H Helset, K Kolnes, B Lødemel, H Ose Velle, S Reite, E Velle. ***Australia*** *(179)* Nambour General Hospital (51): R Grimley, E Ahern, C Cocks, M Courtney, R Devin, J Endacott , C Fawcett , V Harrington, C Johnston, M Koltermann, S Murray, K Ng, G Styles, A Tampiyappa. John Hunter Hospital (29): C Levi, K Chung, L Dark, M Evans, Y Gawarikar, E Kerr, A Loiselle, F Miteff, A Moore, W O'Brien, M Parsons, D Quain, A Royan, M Russell, N Spratt. Gosford Hospital (24): J Sturm, D Crimmins, D Griffiths, P Kavelieros, J Kinsella, A Malhotra, B O'Brien, A Schutz, M Webb, S Whyte, V Zenteno. Westmead Hospital (16): R Lindley, A Bleasel, N Cordato, A Duggins, V Fung, L Gomes, N Ingham, J Ip, P Landau, J Morris, S Vucic. Royal Perth Hospital (15): G Hankey, A Claxton, N Lillywhite. The Canberra Hospital (12): C Lueck, C Andrews, G Danta, C Das, I Harvey, A Hughes, C McColl, A Oon, R Tuck. Royal Brisbane and Women's Hospital (10): S Read, M Badve , M Broad, G Cadigan, H Cavanagh, J Chalk, D Copsinis, K Etherington, R Henderson, R Hull, J O'Sullivan, J Pandian, L Ross-Lee, M Roxas, N Sheikh, G Skinner, A Wong. Austin Health - Repatriation Campus (8): H Dewey, A Brodtmann, G Donnan, A Hughes, M Karonen, H Ma, T Mulcahy, S Petrolo, L Walker, D Young, J Zavala. Nepean Hospital (7): M Thieben, C Harris, M Krause, S Lane, H Park, M Shaffi, J Wood. Box Hill Hospital (7): C Bladin, A Buckland, K Coughlan, B Coulton, A Gilligan, P Lee, S Mullen, Z Ross, P Sien Loh, C Szoeke. ***Portugal*** *(82)* UAVC. Centro Hospitalar de Trás-os-Montes e Alto Douro (42): M Silva, F Afonso, J Gabriel, P Guimarães, A Velon. Hospital Pero da Covilhã (19): M Castelo- Branco, F Alvarez, V Branco, C Coxo, P Goulao, D Leal, S Morgado, R Oliveira, F Paiva, A Rodrigues, M Simoes. Hospital de Santo António (12): G Lopes, T Almeida, M Cardoso, J Chaves, C Correia, M Correia, J Damásio, R Felgueiras, J Pereira, A Tuna. Hospital S.Marcos (9): C Ferreira, E Lourenco, A Machado, R Mare, J Rocha. ***Belgium*** *(73)* Cliniques Universitaires St Luc (73): A Peeters. ***Austria*** *(46)* Landesklinikum Donauregion Tulln (34): K Matz, M Brainin, G Funk, V Reiner-Deitemyer. Krankenhaus Der Barmherzigen Bruder Wien (9): J Ferrari, A Flamm-Horak, G Gruber, R Rattinger. Krankenhaus Göttlicher Heiland (3): W Muellbacher, D Doppelbauer, R Kalchmayr, W Schima, T Wieser, M Zart. ***Switzerland*** *(23)* Universitatsspital Basel (22): P Lyrer, L Bonati, S Engelter, F Fluri, S Muller, E Radue, A Tiemessen, L Walz, F Weisskopf, S Wetzel. Universitätsspital Zürich (1): A Luft, D Fetz, B Hertler, A Pangalu. ***Canada*** *(8)* QEII Health Sciences Centre (8): G Gubitz, P Boulton, J Jarrett, J Moeller, S Phillips. ***Mexico*** *(3)* Instituto Nacional de Neurologia y Neurojrugia MVS (3): A Arauz, L Bermudez, J Calleja, R Garcia.

Supplementary Table 1 Associations between imaging variables and outcomes, adjusted for age, NIHSS and time to randomisation: a) 0-3 hours

	Symptomatic ICH		Death <= 7 days		Death <= 6 months		Alive & Independent OHS 0-2		Favourable Outcome OHS 0-1	
**Imaging variable**	**OR (95% CI)**	**P**	**OR (95% CI)**	**P**	**OR (95% CI)**	**P**	**OR (95% CI)**	**P**	**OR (95% CI)**	**P**
Visible infarct	0.91 (0.44, 1.85)	0.7874	1.44 (0.89, 2.33)	0.1403	1.34 (0.96, 1.87)	0.0868	0.58 (0.39, 0.87)	0.0082	0.53 (0.33, 0.87)	0.0111
Hypoattenuation	0.96 (0.47, 1.95)	0.9077	1.46 (0.90, 2.37)	0.1250	1.34 (0.96, 1.88)	0.0899	0.54 (0.36, 0.82)	0.0035	0.46 (0.28, 0.76)	0.0025
Severe Hypoattenuation	0.86 (0.20, 3.72)	0.8452	1.18 (0.47, 2.94)	0.7298	1.01 (0.52, 1.96)	0.9835	1.01 (0.49, 2.09)	0.9810	0.54 (0.21, 1.41)	0.2094
Large/very large lesion	1.65 (0.76, 3.61)	0.2085	2.39 (1.42, 4.01)	0.0010	1.99 (1.30, 3.06)	0.0016	0.43 (0.23, 0.79)	0.0070	0.48 (0.23, 1.00)	0.0514
Very large lesion	2.07 (0.79, 5.42)	0.1391	3.20 (1.68, 6.08)	0.0004	2.53 (1.36, 4.72)	0.0034	0.30 (0.09, 0.97)	0.0445	0.43 (0.11, 1.63)	0.2145
Swelling	1.41 (0.68, 2.95)	0.3559	1.39 (0.83, 2.31)	0.2064	1.26 (0.87, 1.84)	0.2205	0.54 (0.33, 0.88)	0.0131	0.62 (0.35, 1.09)	0.0954
Hyperattenuated arteries	1.18 (0.56, 2.49)	0.6680	1.41 (0.85, 2.34)	0.1789	1.14 (0.79, 1.65)	0.4804	0.63 (0.39, 1.02)	0.0585	0.62 (0.35, 1.11)	0.1099
Any Leukoaraiosis	1.09 (0.52, 2.28)	0.8196	0.94 (0.57, 1.56)	0.8227	1.32 (0.94, 1.87)	0.1093	0.50 (0.34, 0.73)	0.0003	0.54 (0.35, 0.83)	0.0046
Severe Leukoaraiosis	1.65 (0.81, 3.35)	0.1654	1.14 (0.71, 1.83)	0.5980	1.57 (1.14, 2.17)	0.0062	0.48 (0.33, 0.70)	0.0001	0.54 (0.35, 0.83)	0.0054
Atrophy	0.58 (0.22, 1.51)	0.2609	0.52 (0.25, 1.05)	0.0697	1.22 (0.70, 2.16)	0.4821	0.52 (0.31, 0.88)	0.0149	0.66 (0.37, 1.18)	0.1614
Severe Atrophy	0.88 (0.39, 1.98)	0.7507	0.78 (0.45, 1.37)	0.3885	1.39 (0.97, 2.00)	0.0695	0.86 (0.56, 1.31)	0.4744	0.83 (0.51, 1.36)	0.4601
Old infarct	2.21 (1.09, 4.46)	0.0278	1.39 (0.87, 2.23)	0.1724	1.19 (0.87, 1.65)	0.2768	0.79 (0.55, 1.13)	0.1967	0.82 (0.54, 1.23)	0.3362

Supplementary Table 1 (cont) Associations between imaging variables and outcomes, adjusted for age, NIHSS and time to randomisation: b) 3-4.5 hours

	Symptomatic ICH		Death <= 7 days		Death <= 6 months		Alive & Independent OHS 0-2		Favourable Outcome OHS 0-1	
**Imaging variable**	**OR (95% CI)**	**P**	**OR (95% CI)**	**P**	**OR (95% CI)**	**P**	**OR (95% CI)**	**P**	**OR (95% CI)**	**P**
Visible infarct	1.29 (0.69, 2.42)	0.4175	1.66 (1.09, 2.53)	0.0184	1.31 (0.97, 1.77)	0.0752	0.57 (0.41, 0.78)	0.0004	0.71 (0.50, 1.00)	0.0526
Hypoattenuation	1.33 (0.71, 2.49)	0.3666	1.64 (1.08, 2.49)	0.0212	1.29 (0.96, 1.74)	0.0952	0.58 (0.42, 0.79)	0.0007	0.73 (0.51, 1.04)	0.0772
Severe Hypoattenuation	0.93 (0.28, 3.13)	0.9125	0.54 (0.21, 1.40)	0.2044	0.65 (0.35, 1.21)	0.1748	1.13 (0.61, 2.10)	0.6982	1.47 (0.76, 2.83)	0.2493
Large/very large lesion	1.31 (0.67, 2.57)	0.4298	2.09 (1.35, 3.22)	0.0009	1.92 (1.35, 2.73)	0.0003	0.54 (0.35, 0.84)	0.0067	0.53 (0.31, 0.91)	0.0211
Very large lesion	1.43 (0.65, 3.15)	0.3686	3.01 (1.84, 4.91)	<0.0001	2.04 (1.30, 3.21)	0.0020	0.21 (0.09, 0.48)	0.0002	0.25 (0.09, 0.74)	0.0117
Swelling	0.86 (0.44, 1.70)	0.6683	1.42 (0.92, 2.17)	0.1102	1.42 (1.02, 1.97)	0.0361	0.61 (0.42, 0.90)	0.0114	0.73 (0.48, 1.13)	0.1620
Hyperattenuated arteries	1.37 (0.73, 2.58)	0.3285	1.52 (0.99, 2.31)	0.0530	1.47 (1.06, 2.03)	0.0204	0.43 (0.29, 0.64)	<0.0001	0.61 (0.39, 0.95)	0.0280
Any Leukoaraiosis	0.89 (0.48, 1.68)	0.7271	1.26 (0.82, 1.92)	0.2918	1.55 (1.15, 2.09)	0.0044	0.69 (0.51, 0.94)	0.0177	0.63 (0.45, 0.89)	0.0084
Severe Leukoaraiosis	0.85 (0.44, 1.64)	0.6328	1.17 (0.76, 1.79)	0.4703	1.47 (1.09, 1.98)	0.0111	0.61 (0.44, 0.84)	0.0025	0.67 (0.47, 0.95)	0.0262
Atrophy	1.26 (0.53, 3.02)	0.6040	1.07 (0.60, 1.92)	0.8114	1.49 (0.96, 2.30)	0.0725	0.67 (0.46, 0.98)	0.0388	0.74 (0.49, 1.12)	0.1521
Severe Atrophy	1.00 (0.47, 2.12)	0.9996	0.96 (0.58, 1.58)	0.8642	1.39 (0.99, 1.95)	0.0575	0.82 (0.56, 1.22)	0.3277	0.73 (0.46, 1.14)	0.1647
Old infarct	1.35 (0.74, 2.47)	0.3281	0.72 (0.48, 1.08)	0.1104	0.91 (0.69, 1.21)	0.5226	0.92 (0.69, 1.24)	0.5980	0.74 (0.54, 1.01)	0.0616

Supplementary Table 1 (cont) Associations between imaging variables and outcomes, adjusted for age, NIHSS and time to randomisation: c) 4.5-6 hours

	Symptomatic ICH		Death <= 7 days		Death <= 6 months		Alive & Independent OHS 0-2		Favourable Outcome OHS 0-1	
**Imaging variable**	**OR (95% CI)**	**P**	**OR (95% CI)**	**P**	**OR (95% CI)**	**P**	**OR (95% CI)**	**P**	**OR (95% CI)**	**P**
Visible infarct	2.80 (1.31, 5.99)	0.0078	1.86 (1.06, 3.27)	0.0313	1.61 (1.12, 2.33)	0.0108	0.85 (0.62, 1.16)	0.3094	0.60 (0.43, 0.85)	0.0042
Hypoattenuation	2.85 (1.33, 6.09)	0.0070	1.89 (1.07, 3.33)	0.0271	1.65 (1.14, 2.38)	0.0076	0.85 (0.62, 1.16)	0.3066	0.60 (0.43, 0.85)	0.0044
Severe Hypoattenuation	2.10 (0.78, 5.67)	0.1438	1.86 (0.82, 4.22)	0.1374	1.25 (0.66, 2.38)	0.4940	0.63 (0.34, 1.15)	0.1341	0.49 (0.23, 1.02)	0.0564
Large/very large lesion	0.98 (0.40, 2.42)	0.9680	2.27 (1.27, 4.06)	0.0059	2.41 (1.56, 3.74)	<0.0001	0.53 (0.33, 0.85)	0.0080	0.24 (0.12, 0.48)	<0.0001
Very large lesion	1.06 (0.34, 3.31)	0.9141	3.51 (1.83, 6.76)	0.0002	2.51 (1.41, 4.45)	0.0017	0.37 (0.17, 0.79)	0.0104	0.07 (0.01, 0.50)	0.0083
Swelling	2.05 (0.97, 4.34)	0.0618	2.09 (1.19, 3.66)	0.0099	1.73 (1.15, 2.59)	0.0085	0.59 (0.40, 0.88)	0.0089	0.33 (0.20, 0.56)	<0.0001
Hyperattenuated arteries	2.18 (1.06, 4.48)	0.0337	1.35 (0.77, 2.35)	0.2979	1.74 (1.18, 2.57)	0.0053	0.78 (0.53, 1.14)	0.1981	0.64 (0.41, 1.00)	0.0502
Any Leukoaraiosis	1.15 (0.55, 2.43)	0.7063	1.08 (0.61, 1.93)	0.7880	1.27 (0.87, 1.85)	0.2203	0.98 (0.71, 1.35)	0.8832	0.66 (0.47, 0.93)	0.0174
Severe Leukoaraiosis	1.15 (0.55, 2.41)	0.7113	1.27 (0.71, 2.26)	0.4149	1.21 (0.83, 1.77)	0.3120	0.92 (0.66, 1.29)	0.6341	0.65 (0.45, 0.94)	0.0204
Atrophy	1.09 (0.46, 2.58)	0.8510	0.84 (0.44, 1.60)	0.5919	1.02 (0.65, 1.60)	0.9466	0.96 (0.67, 1.38)	0.8262	0.57 (0.39, 0.82)	0.0026
Severe Atrophy	1.32 (0.55, 3.16)	0.5353	0.83 (0.39, 1.79)	0.6398	0.99 (0.62, 1.57)	0.9515	0.71 (0.47, 1.09)	0.1164	0.71 (0.44, 1.16)	0.1701
Old infarct	1.76 (0.88, 3.51)	0.1105	0.84 (0.48, 1.46)	0.5294	1.12 (0.78, 1.61)	0.5326	0.89 (0.66, 1.20)	0.4445	0.82 (0.60, 1.13)	0.2336

OR=estimated odds of outcome when imaging feature present divided by odds of outcome when feature absent. Estimation was by logistic linear regression, with adjustment for the linear effects of age, NIHSS and time from stroke onset to randomisation

1. Mild/Severe tissue hypoattenuation vs None
2. Severe tissue hypoattenuation vs Mild/None
3. Large /Very Large vs None/Small/Medium
4. Very Large vs None/Small/Medium/Large
5. Mild/Severe Leukoaraiosis vs None
6. Severe Leukoaraiosis vs Mild/None
7. Moderate/Severe Atrophy vs None
8. Severe Atrophy vs Moderate/None

Supplementary Table 2. Comparison of multiple regression models for SICH and OHS 0-2 in three time windows. A) SICH, B) OHS at six months, i) full models, ii) stepwise logistic regression models.

2Ai. Full models of SICH : estimates from models with all imaging variables for three time windows

	0-6 hours		0-4.5 hours		4.5-6 hours	
	OR (95% CI)	P	OR (95% CI)	P	OR (95% CI)	P
Age (yr)	1.00 (0.98, 1.02)	0.9108	0.99 (0.97, 1.02)	0.6515	1.00 (0.97, 1.04)	0.8366
NIHSS	1.06 (1.03, 1.10)	<.0001	1.08 (1.04, 1.11)	<.0001	1.05 (0.99, 1.11)	0.0902
Time to randomisation (hr)	0.98 (0.83, 1.16)	0.8140	1.07 (0.80, 1.41)	0.6550	0.25 (0.10, 0.64)	0.0040
alteplase vs Control	6.65 (3.89, 11.35)	<.0001	6.99 (3.67, 13.33)	<.0001	6.51 (2.45, 17.30)	0.0002
Antiplatelets	1.60 (1.07, 2.38)	0.0212	1.49 (0.93, 2.39)	0.0992	2.12 (0.99, 4.53)	0.0529
Lesion size : Small/medium vs None	1.96 (0.46, 8.34)	0.3641	1.81 (0.37, 8.83)	0.4606	2.23 (0.07, 76.01)	0.6569
Lesion size : Large/Very large vs None	1.91 (0.41, 8.90)	0.4073	2.68 (0.49, 14.59)	0.2549	0.81 (0.02, 30.26)	0.9108
Swelling	0.95 (0.54, 1.69)	0.8667	0.84 (0.41, 1.72)	0.6391	1.61 (0.56, 4.61)	0.3741
Hyperattenuated artery	1.45 (0.92, 2.28)	0.1141	1.29 (0.74, 2.27)	0.3715	1.68 (0.74, 3.81)	0.2160
Hypoattenuation : Mild vs None	0.68 (0.17, 2.78)	0.5883	0.49 (0.11, 2.26)	0.3583	1.19 (0.03, 41.61)	0.9233
Hypoattenuation : Severe vs None	0.71 (0.15, 3.26)	0.6582	0.40 (0.07, 2.29)	0.3043	1.69 (0.04, 69.02)	0.7829
Old lesions	1.75 (1.17, 2.63)	0.0070	1.71 (1.05, 2.78)	0.0321	1.87 (0.87, 4.01)	0.1067
Leukoaraiosis : Mild vs None	1.01 (0.64, 1.59)	0.9748	0.97 (0.57, 1.68)	0.9262	1.08 (0.45, 2.59)	0.8624
Leukoaraiosis : Severe vs None	0.92 (0.51, 1.66)	0.7724	0.94 (0.47, 1.91)	0.8727	0.78 (0.24, 2.53)	0.6815
Atrophy : Mild vs None	0.83 (0.47, 1.48)	0.5315	0.79 (0.39, 1.62)	0.5251	1.00 (0.37, 2.69)	0.9927
Atrophy : Severe vs None	0.82 (0.40, 1.67)	0.5902	0.74 (0.31, 1.77)	0.5036	1.42 (0.39, 5.18)	0.5946

2Aii. Stepwise logistic regression models of SICH : estimates from stepwise logistic models for three time windows

	0-6 hours		0-4.5 hours		4.5-6 hours	
	OR (95% CI)	P	OR (95% CI)	P	OR (95% CI)	P
Age (yr)	0.99 (0.98, 1.01)	0.5567	0.99 (0.97, 1.01)	0.3337	1.01 (0.98, 1.04)	0.6906
NIHSS	1.07 (1.04, 1.10)	<.0001	1.08 (1.05, 1.12)	<.0001	1.05 (1.00, 1.11)	0.0532
Time to randomisation (hr)	0.98 (0.83, 1.15)	0.7987	1.06 (0.80, 1.39)	0.6970	0.26 (0.11, 0.66)	0.0046
alteplase vs Control	6.71 (3.93, 11.46)	<.0001	7.04 (3.70, 13.39)	<.0001	6.49 (2.46, 17.07)	0.0002
Antiplatelets	1.60 (1.07, 2.38)	0.0205			2.14 (1.02, 4.51)	0.0455
Lesion size : Small/medium vs None					3.67 (1.64, 8.22)	0.0016
Lesion size : Large/Very large vs None					1.90 (0.66, 5.49)	0.2331
Hyperattenuated artery	1.61 (1.07, 2.42)	0.0230				
Old lesions	1.67 (1.13, 2.46)	0.0094	1.63 (1.03, 2.59)	0.0364		

The full models for SICH are very similar in the three time windows, while the stepwise selected models appear different and must be interpreted with caution.

2Bi. Full models of OHS 0-2 : estimates from models with all imaging variables for three time windows

	0-6 hours		0-4.5 hours		4.5-6 hours	
	**OR (95% CI)**	**P**	**OR (95% CI)**	**P**	**OR (95% CI)**	**P**
Age (yr)	0.96 (0.96, 0.97)	<.0001	0.96 (0.95, 0.97)	<.0001	0.97 (0.95, 0.98)	<.0001
NIHSS	0.83 (0.82, 0.85)	<.0001	0.84 (0.82, 0.86)	<.0001	0.82 (0.80, 0.85)	<.0001
Time to randomisation (hr)	1.04 (0.96, 1.13)	0.3030	1.00 (0.87, 1.15)	0.9724	1.19 (0.83, 1.71)	0.3305
alteplase vs Control	1.13 (0.94, 1.35)	0.1922	1.03 (0.82, 1.29)	0.7915	1.32 (0.98, 1.78)	0.0660
Lesion size : Small/medium vs None	0.79 (0.37, 1.72)	0.5610	0.64 (0.24, 1.69)	0.3684	1.06 (0.28, 4.02)	0.9365
Lesion size : Large/Very large vs None	0.56 (0.24, 1.27)	0.1653	0.47 (0.17, 1.34)	0.1583	0.68 (0.17, 2.79)	0.5958
Swelling	0.79 (0.56, 1.11)	0.1678	0.90 (0.59, 1.39)	0.6359	0.69 (0.39, 1.21)	0.1936
Hyperattenuated artery	0.70 (0.54, 0.91)	0.0073	0.61 (0.44, 0.86)	0.0043	0.89 (0.58, 1.36)	0.5800
Hypoattenuation : Mild vs None	1.11 (0.52, 2.38)	0.7833	1.12 (0.43, 2.91)	0.8194	1.11 (0.30, 4.09)	0.8706
Hypoattenuation : Severe vs None	1.40 (0.61, 3.21)	0.4322	1.84 (0.65, 5.23)	0.2514	0.91 (0.22, 3.77)	0.8947
Old lesions	0.90 (0.74, 1.09)	0.2788	0.90 (0.71, 1.14)	0.3714	0.87 (0.63, 1.20)	0.3905
Leukoaraiosis : Mild vs None	0.81 (0.65, 1.00)	0.0512	0.72 (0.55, 0.95)	0.0178	1.02 (0.70, 1.48)	0.9105
Leukoaraiosis : Severe vs None	0.64 (0.48, 0.85)	0.0021	0.49 (0.34, 0.69)	<.0001	1.13 (0.68, 1.86)	0.6402
Atrophy : Mild vs None	0.87 (0.67, 1.12)	0.2730	0.76 (0.54, 1.06)	0.1108	1.02 (0.69, 1.53)	0.9072
Atrophy : Severe vs None	0.72 (0.52, 1.00)	0.0519	0.68 (0.45, 1.04)	0.0729	0.70 (0.40, 1.23)	0.2156

2Bii. Stepwise logistic regression models of OHS 0-2 : estimates for three time windows

	0-6 hours		0-4.5 hours		4.5-6 hours	
	**OR (95% CI)**	**P**	**OR (95% CI)**	**P**	**OR (95% CI)**	**P**
Age (yr)	0.96 (0.95, 0.97)	<.0001	0.96 (0.95, 0.97)	<.0001	0.96 (0.95, 0.97)	<.0001
NIHSS	0.83 (0.82, 0.85)	<.0001	0.84 (0.82, 0.86)	<.0001	0.82 (0.80, 0.85)	<.0001
Time to randomisation (hr)	1.04 (0.96, 1.13)	0.2881	1.00 (0.87, 1.14)	0.9491	1.21 (0.84, 1.72)	0.3027
rtPA vs Control	1.12 (0.94, 1.34)	0.1965	1.04 (0.83, 1.30)	0.7366	1.30 (0.97, 1.75)	0.0779
Lesion size : Small/medium vs None	0.85 (0.67, 1.07)	0.1731	0.76 (0.56, 1.03)	0.0725		
Lesion size : Large/Very large vs None	0.54 (0.39, 0.74)	0.0001	0.52 (0.35, 0.77)	0.0011		
Swelling					0.59 (0.40, 0.88)	0.0094
Hyperattenuated artery	0.71 (0.55, 0.92)	0.0085	0.62 (0.44, 0.86)	0.0044		
Leukoaraiosis : Mild vs None	0.76 (0.62, 0.93)	0.0092	0.66 (0.51, 0.86)	0.0021		
Leukoaraiosis : Severe vs None	0.59 (0.45, 0.79)	0.0003	0.45 (0.32, 0.64)	<.0001		

The models selected by stepwise methods differ, indicating that conclusions should not be based on the stepwise approach.

Supplementary Table 3. Unadjusted and adjusted subgroup effects on ordinal outcome (5 levels: 0, 1, 2, 3, 4-6) at six months, Odds ratios and 99% Confidence Intervals.

	**OHS 0-2/Total (%)**		**Ordinal outcome Unadjusted**		**Ordinal outcome Adjusted**	
	**alteplase**	**Control**	**OR (99% CI)**	**P**	**OR (99% CI)**	**P**
**Age**				.		.
<= 80 years	328/693 (47.3%)	343/715 (48.0%)	1.08 (0.84, 1.38)	0.1709	1.08 (0.84, 1.40)	0.0398
> 80 years	222/814 (27.3%)	185/795 (23.3%)	1.30 (1.01, 1.68)	.	1.45 (1.10, 1.92)	.
**Sex**				.		.
Female	237/778 (30.5%)	232/783 (29.6%)	1.16 (0.91, 1.49)	0.9673	1.36 (1.04, 1.79)	0.3415
Male	313/729 (42.9%)	296/727 (40.7%)	1.17 (0.91, 1.49)	.	1.18 (0.90, 1.53)	.
**NIHSS score**				.		.
0 to 5	220/303 (72.6%)	229/304 (75.3%)	1.11 (0.77, 1.62)	0.0162	1.12 (0.77, 1.63)	0.0118
6 to 15	273/723 (37.8%)	266/723 (36.8%)	1.13 (0.89, 1.45)	.	1.18 (0.92, 1.52)	.
16 to 24	50/400 (12.5%)	32/418 (7.7%)	1.57 (1.00, 2.47)	.	1.65 (1.03, 2.64)	.
>= 25	7/81 (8.6%)	1/65 (1.5%)	4.13 (0.92, 18.6)	.	4.79 (1.01, 22.8)	.
**Time to randomisation**				.		.
≤3 hours	132/431 (30.6%)	94/415 (22.7%)	1.45 (1.02, 2.06)	0.4422	1.50 (1.03, 2.20)	0.6117
3-4.5 hours	182/575 (31.7%)	224/596 (37.6%)	0.97 (0.73, 1.29)	.	1.08 (0.79, 1.46)	.
> 4.5 hours	236/501 (47.1%)	210/497 (42.3%)	1.23 (0.92, 1.65)	.	1.33 (0.97, 1.81)	.
**Acute ischaemic change on randomisation scan according to expert panel**						
No	392/883 (44.4%)	379/910 (41.6%)	1.22 (0.98, 1.52)	0.4864	1.32 (1.04, 1.67)	0.4435
Yes	158/624 (25.3%)	149/600 (24.8%)	1.11 (0.83, 1.48)	.	1.19 (0.86, 1.64)	.
**Treatment with antiplatelet drugs in previous 48 hours**						
No	286/732 (39.1%)	278/720 (38.6%)	1.16 (0.90, 1.49)	0.9751	1.25 (0.95, 1.63)	0.8727
Yes	263/771 (34.1%)	249/783 (31.8%)	1.16 (0.90, 1.48)	.	1.27 (0.97, 1.65)	.
**Early ischaemic lesion swelling**.						
No	468/1152 (40.6%)	463/1171 (39.5%)	1.17 (0.96, 1.42)	0.9919	1.27 (1.03, 1.57)	0.8848
Yes	82/355 (23.1%)	65/339 (19.2%)	1.18 (0.79, 1.75)	.	1.23 (0.79, 1.92)	.
**Early ischaemic issue hypoattenuation**.						
No	395/892 (44.3%)	383/922 (41.5%)	1.22 (0.98, 1.52)	0.5025	1.31 (1.04, 1.66)	0.4752
Yes	155/615 (25.2%)	145/588 (24.7%)	1.11 (0.83, 1.49)	.	1.19 (0.86, 1.65)	.
**Hyperattenuated arteries**.						
No	475/1131 (42.0%)	452/1151 (39.3%)	1.23 (1.01, 1.50)	0.1628	1.32 (1.07, 1.64)	0.2088
Yes	75/376 (19.9%)	76/359 (21.2%)	0.97 (0.66, 1.43)	.	1.06 (0.69, 1.62)	.
**Early ischaemic lesion size**[1].						
None visible	394/885 (44.5%)	380/914 (41.6%)	1.22 (0.98, 1.53)	0.8061	1.31 (1.04, 1.66)	0.7198

	OHS 0-2/Total (%)		Ordinal outcome Unadjusted		Ordinal outcome Adjusted	
	**alteplase**	**Control**	**OR (99% CI)**	**P**	**OR (99% CI)**	**P**
Small/ Medium	107/360 (29.7%)	108/347 (31.1%)	1.08 (0.75, 1.56)	.	1.16 (0.78, 1.73)	.
Large	36/124 (29.0%)	31/137 (22.6%)	1.27 (0.68, 2.39)	.	1.27 (0.63, 2.55)	.
Very large	13/138 (9.4%)	9/112 (8.0%)	1.34 (0.58, 3.07)	.	1.65 (0.63, 4.30)	.
**Early ischaemic lesion large**						
No	537/1369 (39.2%)	519/1398 (37.1%)	1.19 (0.99, 1.42)	0.7588	1.27 (1.05, 1.55)	0.7648
Yes	13/138 (9.4%)	9/112 (8.0%)	1.34 (0.58, 3.07)	.	1.65 (0.63, 4.30)	.
**Old infarcts**				.		.
No	326/822 (39.7%)	311/859 (36.2%)	1.27 (1.00, 1.60)	0.1937	1.35 (1.05, 1.73)	0.3706
Yes	224/685 (32.7%)	217/651 (33.3%)	1.06 (0.81, 1.38)	.	1.17 (0.88, 1.57)	.
**Atrophy**				.		.
No	176/346 (50.9%)	172/344 (50.0%)	1.22 (0.86, 1.73)	0.7169	1.42 (0.98, 2.06)	0.3292
Yes	374/1161 (32.2%)	356/1166 (30.5%)	1.14 (0.93, 1.40)	.	1.21 (0.97, 1.51)	.
**Leukoaraiosis**				.		.
No	319/742 (43.0%)	316/728 (43.4%)	1.10 (0.86, 1.40)	0.4138	1.23 (0.94, 1.59)	0.7332
Yes	231/765 (30.2%)	212/782 (27.1%)	1.23 (0.95, 1.58)	.	1.28 (0.97, 1.69)	.
**Combination of large lesion, hyperattenuated arteries and leukoaraiosis**						
None	268/519 (51.6%)	260/524 (49.6%)	1.23 (0.92, 1.63)	0.6798	1.36 (1.00, 1.84)	0.5518
One out of three	248/733 (33.8%)	240/758 (31.7%)	1.18 (0.92, 1.51)	.	1.21 (0.92, 1.58)	.
Two out of three	31/219 (14.2%)	27/189 (14.3%)	1.10 (0.61, 1.98)	.	1.27 (0.67, 2.41)	.
All three	3/36 (8.3%)	1/39 (2.6%)	1.44 (0.27, 7.74)	.	2.84 (0.33, 24.7)	.

[1] Lesion size refers to the extent of the acute ischemic tissue change according to the IST-3 score (see Methods, main paper).[1]

Supplementary Table 4. A) Adjusted effect of treatment with alteplase on SICH, randomisation 0-3 hours, Odds Ratio and 99% Confidence Interval

	**sICH/Total (%)**		**Unadjusted**		**Adjusted [1]**	
	**alteplase**	**Control**	**OR (99% CI)**	**P [2]**	**OR (99% CI)**	**P [2]**
**All**	32/431 (7.4%)	4/415 (1.0%)	8.24 (2.08, 32.7)		8.77 (2.20, 35.0)	
**Acute ischemic change on randomisation scan according to expert panel**						
**No**	19/264 (7.2%)	2/258 (0.8%)	9.92 (1.44, 68.2)	0.70	10.31 (1.49, 71.5)	0.75
**Yes**	13/167 (7.8%)	2/157 (1.3%)	6.54 (0.90, 47.3)		7.15 (0.97, 52.6)	
**Treatment with antiplatelet drugs in previous 48 hours**						
**No**	9/192 (4.7%)	2/179 (1.1%)	4.35 (0.57, 33.2)	0.33	4.32 (0.56, 33.1)	0.32
**Yes**	23/239 (9.6%)	2/236 (0.8%)	12.46 (1.84, 84.5)		14.43 (2.09, 99.9)	
**Swelling**						
**No**	21/318 (6.6%)	3/330 (0.9%)	7.71 (1.55, 38.3)	0.89	8.07 (1.61, 40.4)	0.85
**Yes**	11/113 (9.7%)	1/85 (1.2%)	9.05 (0.60, 137)		9.81 (0.64, 152)	
**Tissue density change**						
**No**	19/269 (7.1%)	2/262 (0.8%)	9.88 (1.44, 67.9)	0.71	10.10 (1.46, 69.9)	0.78
**Yes**	13/162 (8.0%)	2/153 (1.3%)	6.59 (0.91, 47.7)		7.29 (0.99, 53.7)	
**Hyperdense arteries**						
**No**	21/329 (6.4%)	4/315 (1.3%)	5.30 (1.28, 21.9)	0.97	5.50 (1.32, 23.0)	0.97
**Yes**	11/102 (10.8%)	0/100 (0.0%)				
**Lesion size**						
**None visible**	19/264 (7.2%)	2/259 (0.8%)	9.96 (1.45, 68.5)	0.75	10.39 (1.50, 72.1)	0.80
**Small/Medium**	4/98 (4.1%)	1/86 (1.2%)	3.62 (0.20, 66.1)		4.16 (0.21, 80.8)	
**Large**	4/34 (11.8%)	0/44 (0.0%)				
**Very large**	5/35 (14.3%)	1/26 (3.8%)	4.17 (0.23, 76.2)		4.88 (0.24, 98.7)	
**Large/ Very large lesion**						
**No**	23/362 (6.4%)	3/345 (0.9%)	7.73 (1.57, 38.1)	0.81	8.08 (1.63, 40.0)	0.81
**Yes**	9/69 (13.0%)	1/70 (1.4%)	10.35 (0.66, 162)		10.58 (0.66, 169)	
**Old lesion**						
**No**	11/219 (5.0%)	2/224 (0.9%)	5.87 (0.80, 43.2)	0.60	6.35 (0.85, 47.5)	0.67
**Yes**	21/212 (9.9%)	2/191 (1.0%)	10.38 (1.52, 71.1)		10.86 (1.57, 75.3)	
**Atrophy**						
**No**	6/67 (9.0%)	1/49 (2.0%)	4.72 (0.28, 79.7)	0.59	4.50 (0.26, 79.4)	0.51
**Yes**	26/364 (7.1%)	3/366 (0.8%)	9.30 (1.91, 45.3)		10.00 (2.04, 49.1)	
**White matter lesion**						
**No**	11/163 (6.7%)	2/161 (1.2%)	5.75 (0.78, 42.6)	0.56	6.08 (0.80, 46.0)	0.52
**Yes**	21/268 (7.8%)	2/254 (0.8%)	10.71 (1.57, 73.1)		11.47 (1.67, 78.9)	
**Combination of hyperdense arteries and old lesion**						
**No old lesion or hyperdense arteries**	8/162 (4.9%)	2/157 (1.3%)	4.03 (0.51, 31.5)	0.17	4.07 (0.50, 33.1)	0.19
**Either old lesion or hyperdense arteries**	16/224 (7.1%)	2/225 (0.9%)	8.58 (1.22, 60.1)		9.56 (1.35, 68.0)	
**Both old lesions and hyperdense arteries**	8/45 (17.8%)	0/33 (0.0%)				

*. Adjusted for age, NIHSS and delay to randomisation (all linear); 2 P for interaction of rtPA/Control with subgroup factor

Supplementary Table 4. B) Adjusted* subgroup effects on SICH by 7 days: Patients randomised 3-4.5 hours, Odds Ratio, 99% CI

	sICH/Total (%)		Unadjusted		Adjusted	
	alteplase	Control	OR (99% CI)	P	OR (99% CI)	P
**All**	40/575 (7.0%)	7/596 (1.2%)	6.29 (2.17, 18.3)		6.11 (2.09, 17.9)	
**Acute ischemic change on randomisation scan according to expert panel**						
**No**	17/317 (5.4%)	3/362 (0.8%)	6.78 (1.33, 34.5)	0.82	7.09 (1.37, 36.6)	0.80
**Yes**	23/258 (8.9%)	4/234 (1.7%)	5.63 (1.37, 23.2)		5.54 (1.33, 23.0)	
**Treatment with antiplatelet drugs in previous 48 hours**						
**No**	16/291 (5.5%)	4/281 (1.4%)	4.03 (0.94, 17.3)	0.30	3.92 (0.90, 17.1)	0.31
**Yes**	24/284 (8.5%)	3/315 (1.0%)	9.60 (1.95, 47.2)		9.47 (1.91, 46.9)	
**Swelling**						
**No**	30/440 (6.8%)	4/457 (0.9%)	8.29 (2.08, 33.0)	0.34	8.11 (2.02, 32.5)	0.35
**Yes**	10/135 (7.4%)	3/139 (2.2%)	3.63 (0.65, 20.4)		3.64 (0.64, 20.8)	
**Tissue density change**						
**No**	17/320 (5.3%)	3/367 (0.8%)	6.81 (1.34, 34.6)	0.81	7.15 (1.39, 36.9)	0.78
**Yes**	23/255 (9.0%)	4/229 (1.7%)	5.58 (1.35, 23.0)		5.47 (1.32, 22.7)	
**Hyperdense arteries**						
**No**	25/415 (6.0%)	3/454 (0.7%)	9.63 (1.98, 46.9)	0.24	9.71 (1.98, 47.6)	0.22
**Yes**	15/160 (9.4%)	4/142 (2.8%)	3.57 (0.81, 15.7)		3.52 (0.78, 15.9)	
**Lesion size**						
**None visible**	17/317 (5.4%)	3/364 (0.8%)	6.82 (1.34, 34.6)	0.63	7.14 (1.38, 36.8)	0.58
**Small/Medium**	11/138 (8.0%)	2/136 (1.5%)	5.80 (0.78, 43.1)		5.98 (0.80, 44.9)	
**Large**	5/59 (8.5%)	0/44 (0.0%)				
**Very large**	7/61 (11.5%)	2/52 (3.8%)	3.24 (0.39, 27.2)		2.69 (0.30, 24.4)	
**Large/ Very large lesion**						
**No**	28/455 (6.2%)	5/500 (1.0%)	6.49 (1.84, 22.9)	0.81	6.57 (1.85, 23.4)	0.76
**Yes**	12/120 (10.0%)	2/96 (2.1%)	5.22 (0.71, 38.6)		5.00 (0.67, 37.5)	
**Old lesion**						
**No**	19/308 (6.2%)	3/328 (0.9%)	7.12 (1.42, 35.8)	0.78	7.24 (1.42, 36.9)	0.75
**Yes**	21/267 (7.9%)	4/268 (1.5%)	5.63 (1.36, 23.4)		5.51 (1.31, 23.1)	
**Atrophy**						
**No**	6/129 (4.7%)	3/136 (2.2%)	2.16 (0.34, 13.7)	0.10	2.15 (0.33, 13.8)	0.14
**Yes**	34/446 (7.6%)	4/460 (0.9%)	9.40 (2.38, 37.1)		8.79 (2.21, 35.0)	
**White matter lesion**						
**No**	20/303 (6.6%)	5/291 (1.7%)	4.04 (1.10, 14.9)	0.23	4.12 (1.09, 15.5)	0.25
**Yes**	20/272 (7.4%)	2/305 (0.7%)	12.02 (1.76, 82.2)		11.99 (1.74, 82.6)	
**Combination of hyperdense arteries and old lesion**						
**No old lesion or hyperdense arteries**	10/206 (4.9%)	2/229 (0.9%)	5.79 (0.78, 43.3)	0.23	6.04 (0.80, 45.8)	0.22
**Either old lesion or hyperdense arteries**	24/311 (7.7%)	2/324 (0.6%)	13.46 (2.00, 90.7)		13.43 (1.98, 91.1)	
**Both old lesions and hyperdense arteries**	6/58 (10.3%)	3/43 (7.0%)	1.54 (0.23, 10.3)		1.59 (0.23, 11.2)	

*. Adjusted for age, NIHSS and delay to randomisation (all linear); 2 P for interaction of rtPA/Control with subgroup factor

Supplementary Table 4. C) Adjusted* subgroup effects on SICH by 7 days: Patients randomised 4.5-6 hours, Odds Ratio, 99% CI

	sICH Total (%)		Unadjusted		Adjusted	
	**alteplase**	**Control**	**OR (99% CI)**	**P**	**OR (99% CI)**	**P**
**All**	30/501 (6.0%)	5/497 (1.0%)	6.27 (1.79, 22.0)		6.41 (1.81, 22.7)	
**Acute ischemic change on randomisation scan according to expert panel**						
**No**	11/302 (3.6%)	0/290 (0.0%)				
**Yes**	19/199 (9.5%)	5/207 (2.4%)	4.26 (1.14, 16.0)		4.01 (1.05, 15.2)	
**Treatment with antiplatelet drugs in previous 48 hours**						
**No**	8/253 (3.2%)	4/266 (1.5%)	2.14 (0.43, 10.5)	0.05	2.16 (0.43, 10.8)	0.05
**Yes**	22/248 (8.9%)	1/231 (0.4%)	22.39 (1.59, 315)		23.90 (1.68, 340)	
**Swelling**						
**No**	19/394 (4.8%)	2/384 (0.5%)	9.67 (1.41, 66.2)	0.40	10.03 (1.46, 69.1)	0.37
**Yes**	11/107 (10.3%)	3/113 (2.7%)	4.20 (0.76, 23.4)		3.97 (0.70, 22.7)	
**Tissue density change**						
**No**	11/303 (3.6%)	0/293 (0.0%)				
**Yes**	19/198 (9.6%)	5/204 (2.5%)	4.22 (1.13, 15.8)		3.97 (1.04, 15.1)	
**Hyperdense arteries**						
**No**	16/387 (4.1%)	4/381 (1.0%)	4.06 (0.95, 17.4)	0.25	4.29 (1.00, 18.5)	0.31
**Yes**	14/114 (12.3%)	1/116 (0.9%)	16.10 (1.09, 237)		14.72 (0.98, 221)	
**Lesion size**						
**None visible**	11/304 (3.6%)	0/291 (0.0%)				
**Small/Medium**	14/124 (11.3%)	3/124 (2.4%)	5.13 (0.96, 27.4)		5.03 (0.91, 27.8)	
**Large**	2/31 (6.5%)	1/49 (2.0%)	3.31 (0.13, 82.2)		3.84 (0.12, 123)	
**Very large**	3/42 (7.1%)	1/33 (3.0%)	2.46 (0.12, 51.3)		2.03 (0.09, 45.4)	
**Large/ Very large lesion**						
**No**	25/428 (5.8%)	3/415 (0.7%)	8.52 (1.75, 41.5)	0.31	9.01 (1.83, 44.4)	0.23
**Yes**	5/73 (6.8%)	2/82 (2.4%)	2.94 (0.33, 26.5)		2.58 (0.28, 24.1)	
**Old lesion**						
**No**	12/295 (4.1%)	4/305 (1.3%)	3.19 (0.71, 14.3)	0.14	3.16 (0.70, 14.3)	0.13
**Yes**	18/206 (8.7%)	1/192 (0.5%)	18.29 (1.28, 261)		20.56 (1.40, 301)	
**Atrophy**						
**No**	7/150 (4.7%)	2/158 (1.3%)	3.82 (0.47, 30.8)	0.48	3.69 (0.45, 30.1)	0.42
**Yes**	23/351 (6.6%)	3/339 (0.9%)	7.85 (1.60, 38.6)		8.46 (1.70, 42.2)	
**White matter lesion**						
**No**	14/276 (5.1%)	3/275 (1.1%)	4.84 (0.93, 25.3)	0.58	4.85 (0.92, 25.6)	0.57
**Yes**	16/225 (7.1%)	2/222 (0.9%)	8.42 (1.20, 59.0)		8.98 (1.26, 63.8)	
**Combination of hyperdense arteries and old lesion**						
**No old lesion or hyperdense arteries**	8/222 (3.6%)	3/218 (1.4%)	2.68 (0.46, 15.6)	0.05	2.79 (0.48, 16.4)	0.05
**Either old lesion or hyperdense arteries**	12/238 (5.0%)	2/250 (0.8%)	6.58 (0.91, 47.8)		5.83 (0.79, 43.1)	
**Both old lesions and hyperdense arteries**	10/41 (24.4%)	0/29 (0.0%)				

*. Adjusted for age, NIHSS and delay to randomisation (all linear); 2 P for interaction of rtPA/Control with subgroup factor

Supplementary Table 4. D) Adjusted* effect of treatment on the primary outcome (OHS 0-2), 0-3 hours, Odds Ratio, 99% CI

	**Alive & Independent Total (%)**		**Unadjusted**		**Adjusted**	
	**alteplase**	**Control**	**OR (99% CI)**	**P**	**OR (99% CI)**	**P**
**All**	132/431 (30.6%)	94/415 (22.7%)	1.51 (1.01, 2.26)		1.66 (1.03, 2.65)	
**Acute ischemic change on randomisation scan according to expert panel**						
**No**	101/264 (38.3%)	70/258 (27.1%)	1.66 (1.02, 2.71)	0.43	2.14 (1.21, 3.79)	0.05
**Yes**	31/167 (18.6%)	24/157 (15.3%)	1.26 (0.59, 2.72)		0.95 (0.40, 2.29)	
**Treatment with antiplatelet drugs in previous 48 hours**						
**No**	59/192 (30.7%)	52/179 (29.1%)	1.08 (0.60, 1.94)	0.05	1.27 (0.63, 2.56)	0.15
**Yes**	73/239 (30.5%)	42/236 (17.8%)	2.03 (1.15, 3.59)		2.08 (1.10, 3.95)	
**Swelling**						
**No**	110/318 (34.6%)	84/330 (25.5%)	1.55 (0.99, 2.42)	0.72	1.84 (1.10, 3.10)	0.45
**Yes**	22/113 (19.5%)	10/85 (11.8%)	1.81 (0.63, 5.24)		1.28 (0.38, 4.33)	
**Tissue density change**						
**No**	103/269 (38.3%)	71/262 (27.1%)	1.67 (1.03, 2.70)	0.40	2.19 (1.24, 3.87)	0.03
**Yes**	29/162 (17.9%)	23/153 (15.0%)	1.23 (0.56, 2.71)		0.89 (0.36, 2.20)	
**Hyperdense arteries**						
**No**	115/329 (35.0%)	79/315 (25.1%)	1.61 (1.03, 2.51)	0.41	1.99 (1.17, 3.38)	0.06
**Yes**	17/102 (16.7%)	15/100 (15.0%)	1.13 (0.42, 3.06)		0.84 (0.27, 2.57)	
**Lesion size**						
**None visible**	101/264 (38.3%)	70/259 (27.0%)	1.67 (1.03, 2.72)	0.62	2.14 (1.21, 3.79)	0.16
**Small/Medium**	21/98 (21.4%)	18/86 (20.9%)	1.03 (0.41, 2.62)		0.65 (0.22, 1.97)	
**Large**	8/34 (23.5%)	4/44 (9.1%)	3.08 (0.56, 16.9)		3.54 (0.51, 24.5)	
**Very large**	2/35 (5.7%)	2/26 (7.7%)	0.73 (0.05, 10.5)		11.62 (0.01, 1E4)	
**Large/ Very large lesion**						
**No**	122/362 (33.7%)	88/345 (25.5%)	1.48 (0.97, 2.28)	0.73	1.64 (1.00, 2.70)	0.91
**Yes**	10/69 (14.5%)	6/70 (8.6%)	1.81 (0.44, 7.40)		1.76 (0.37, 8.44)	
**Old lesion**						
**No**	73/219 (33.3%)	53/224 (23.7%)	1.61 (0.93, 2.79)	0.67	1.74 (0.90, 3.37)	0.86
**Yes**	59/212 (27.8%)	41/191 (21.5%)	1.41 (0.77, 2.57)		1.60 (0.81, 3.14)	
**Atrophy**						
**No**	32/67 (47.8%)	24/49 (49.0%)	0.95 (0.36, 2.51)	0.21	1.72 (0.47, 6.28)	0.93
**Yes**	100/364 (27.5%)	70/366 (19.1%)	1.60 (1.01, 2.53)		1.63 (0.98, 2.72)	
**White matter lesion**						
**No**	67/163 (41.1%)	55/161 (34.2%)	1.35 (0.74, 2.43)	0.40	1.40 (0.66, 2.95)	0.33
**Yes**	65/268 (24.3%)	39/254 (15.4%)	1.77 (0.99, 3.15)		1.92 (1.02, 3.63)	
**Combination of large/very large lesion, hyperdense arteries and leukoaraiosis**						
**None**	54/106 (50.9%)	44/110 (40.0%)	1.56 (0.77, 3.16)	0.97	1.81 (0.73, 4.50)	0.75
**One out of three**	65/232 (28.0%)	41/213 (19.2%)	1.63 (0.91, 2.93)		1.77 (0.93, 3.38)	
**Two out of three**	12/72 (16.7%)	8/65 (12.3%)	1.42 (0.40, 5.07)		1.24 (0.32, 4.85)	
**All three**	1/21 (4.8%)	1/27 (3.7%)	1.30 (0.03, 53.8)			

*. Adjusted for age, NIHSS and delay to randomisation (all linear); 2 P for interaction of rtPA/Control with subgroup factor

Supplementary Table 4. E) Adjusted* effect of treatment on the primary outcome (OHS 0-2), 3-4.5 hours, Odds Ratio, 99% CI

	**Alive & Independent Total (%)**		**Unadjusted**		**Adjusted**	
	**alteplase**	**Control**	**OR (99% CI)**	**P**	**OR (99% CI)**	**P**
**All**	182/575 (31.7%)	224/596 (37.6%)	0.77 (0.56, 1.06)		0.74 (0.51, 1.07)	
**Acute ischemic change on randomisation scan according to expert panel**						
**No**	126/317 (39.7%)	167/362 (46.1%)	0.77 (0.52, 1.15)	0.67	0.70 (0.44, 1.12)	0.52
**Yes**	56/258 (21.7%)	57/234 (24.4%)	0.86 (0.50, 1.50)		0.87 (0.45, 1.67)	
**Treatment with antiplatelet drugs in previous 48 hours**						
**No**	101/291 (34.7%)	117/281 (41.6%)	0.75 (0.48, 1.16)	0.87	0.64 (0.37, 1.11)	0.40
**Yes**	81/284 (28.5%)	107/315 (34.0%)	0.78 (0.49, 1.22)		0.81 (0.48, 1.38)	
**Swelling**						
**No**	153/440 (34.8%)	195/457 (42.7%)	0.72 (0.50, 1.02)	0.26	0.67 (0.44, 1.01)	0.15
**Yes**	29/135 (21.5%)	29/139 (20.9%)	1.04 (0.48, 2.22)		1.21 (0.48, 3.04)	
**Tissue density change**						
**No**	127/320 (39.7%)	168/367 (45.8%)	0.78 (0.52, 1.16)	0.75	0.71 (0.44, 1.13)	0.54
**Yes**	55/255 (21.6%)	56/229 (24.5%)	0.85 (0.49, 1.48)		0.86 (0.45, 1.67)	
**Hyperdense arteries**						
**No**	159/415 (38.3%)	194/454 (42.7%)	0.83 (0.58, 1.19)	0.40	0.76 (0.50, 1.16)	0.72
**Yes**	23/160 (14.4%)	30/142 (21.1%)	0.63 (0.29, 1.38)		0.67 (0.27, 1.68)	
**Lesion size**						
**None visible**	126/317 (39.7%)	167/364 (45.9%)	0.78 (0.52, 1.16)	0.61	0.71 (0.44, 1.13)	0.36
**Small/Medium**	35/138 (25.4%)	40/136 (29.4%)	0.82 (0.41, 1.64)		0.74 (0.32, 1.67)	
**Large**	17/59 (28.8%)	13/44 (29.5%)	0.97 (0.31, 2.98)		0.93 (0.23, 3.81)	
**Very large**	4/61 (6.6%)	4/52 (7.7%)	0.84 (0.13, 5.57)		1.15 (0.15, 8.89)	
**Large/ Very large lesion**						
**No**	161/455 (35.4%)	207/500 (41.4%)	0.78 (0.55, 1.09)	0.53	0.71 (0.47, 1.06)	0.30
**Yes**	21/120 (17.5%)	17/96 (17.7%)	0.99 (0.39, 2.49)		1.14 (0.38, 3.41)	
**Old lesion**						
**No**	108/308 (35.1%)	126/328 (38.4%)	0.87 (0.57, 1.32)	0.29	0.82 (0.49, 1.37)	0.39
**Yes**	74/267 (27.7%)	98/268 (36.6%)	0.67 (0.41, 1.08)		0.64 (0.37, 1.13)	
**Atrophy**						
**No**	61/129 (47.3%)	74/136 (54.4%)	0.75 (0.40, 1.42)	0.94	0.67 (0.30, 1.51)	0.68
**Yes**	121/446 (27.1%)	150/460 (32.6%)	0.77 (0.53, 1.12)		0.77 (0.50, 1.19)	
**White matter lesion**						
**No**	114/303 (37.6%)	134/291 (46.0%)	0.71 (0.46, 1.09)	0.63	0.67 (0.40, 1.12)	0.55
**Yes**	68/272 (25.0%)	90/305 (29.5%)	0.80 (0.49, 1.29)		0.79 (0.45, 1.39)	
**Combination of large/very large lesion, hyperdense arteries and leukoaraiosis**						
**None**	87/187 (46.5%)	106/195 (54.4%)	0.73 (0.43, 1.24)	0.86	0.66 (0.35, 1.22)	0.51
**One out of three**	79/251 (31.5%)	100/284 (35.2%)	0.85 (0.53, 1.36)		0.77 (0.45, 1.34)	
**Two out of three**	15/110 (13.6%)	17/92 (18.5%)	0.70 (0.26, 1.88)		0.85 (0.27, 2.61)	
**All three**	1/27 (3.7%)	1/25 (4.0%)	0.92 (0.02, 37.9)			

*. Adjusted for age, NIHSS and delay to randomisation (all linear); 2 P for interaction of rtPA/Control with subgroup factor

Supplementary Table 4. F) Adjusted* effect of treatment on the primary outcome (OHS 0-2), 4.5-6 hours, Odds Ratio, 99% CI

	Alive & Independent Total (%)		Unadjusted		Adjusted	
	**alteplase**	**Control**	**OR (99% CI)**	**P**	**OR (99% CI)**	**P**
**All**	236/501 (47.1%)	210/497 (42.3%)	1.22 (0.88, 1.69)		1.31 (0.89, 1.93)	
**Acute ischemic change on randomisation scan according to expert panel**						
**No**	165/302 (54.6%)	142/290 (49.0%)	1.26 (0.82, 1.92)	0.70	1.29 (0.80, 2.07)	0.98
**Yes**	71/199 (35.7%)	68/207 (32.9%)	1.13 (0.66, 1.94)		1.36 (0.69, 2.67)	
**Swelling**						
**No**	205/394 (52.0%)	184/384 (47.9%)	1.18 (0.81, 1.71)	0.67	1.25 (0.82, 1.91)	0.55
**Yes**	31/107 (29.0%)	26/113 (23.0%)	1.36 (0.62, 3.02)		1.63 (0.61, 4.35)	
**Tissue density change**						
**No**	165/303 (54.5%)	144/293 (49.1%)	1.24 (0.81, 1.89)	0.83	1.26 (0.78, 2.02)	0.80
**Yes**	71/198 (35.9%)	66/204 (32.4%)	1.17 (0.68, 2.01)		1.42 (0.72, 2.80)	
**Hyperdense arteries**						
**No**	201/387 (51.9%)	179/381 (47.0%)	1.22 (0.84, 1.77)	0.99	1.23 (0.80, 1.89)	0.45
**Yes**	35/114 (30.7%)	31/116 (26.7%)	1.21 (0.57, 2.58)		1.78 (0.71, 4.45)	
**Lesion size**						
**None visible**	167/304 (54.9%)	143/291 (49.1%)	1.26 (0.83, 1.93)	0.99	1.30 (0.81, 2.09)	1.00
**Small/Medium**	51/124 (41.1%)	50/124 (40.3%)	1.03 (0.53, 2.01)		1.32 (0.58, 2.99)	
**Large**	11/31 (35.5%)	14/49 (28.6%)	1.38 (0.39, 4.87)		1.29 (0.25, 6.57)	
**Very large**	7/42 (16.7%)	3/33 (9.1%)	2.00 (0.30, 13.2)		1.78 (0.13, 24.2)	
**Large/ Very large lesion**						
**No**	218/428 (50.9%)	193/415 (46.5%)	1.19 (0.84, 1.70)	0.91	1.30 (0.86, 1.95)	0.97
**Yes**	18/73 (24.7%)	17/82 (20.7%)	1.25 (0.46, 3.37)		1.25 (0.35, 4.46)	
**Old lesion**						
**No**	145/295 (49.2%)	132/305 (43.3%)	1.27 (0.83, 1.93)	0.73	1.28 (0.78, 2.12)	0.91
**Yes**	91/206 (44.2%)	78/192 (40.6%)	1.16 (0.69, 1.95)		1.34 (0.73, 2.46)	
**Atrophy**						
**No**	83/150 (55.3%)	74/158 (46.8%)	1.41 (0.78, 2.54)	0.47	1.39 (0.68, 2.81)	0.76
**Yes**	153/351 (43.6%)	136/339 (40.1%)	1.15 (0.77, 1.72)		1.29 (0.81, 2.05)	
**White matter lesion**						
**No**	138/276 (50.0%)	127/275 (46.2%)	1.17 (0.75, 1.81)	0.69	1.23 (0.73, 2.06)	0.74
**Yes**	98/225 (43.6%)	83/222 (37.4%)	1.29 (0.79, 2.13)		1.40 (0.78, 2.51)	
**Combination of large/very large lesion, hyperdense arteries and leukoaraiosis**						
**None**	108/193 (56.0%)	101/186 (54.3%)	1.07 (0.63, 1.82)	0.71	1.10 (0.60, 2.03)	0.53
**One out of three**	108/220 (49.1%)	90/220 (40.9%)	1.39 (0.85, 2.29)		1.47 (0.84, 2.59)	
**Two out of three**	17/72 (23.6%)	16/73 (21.9%)	1.10 (0.40, 3.06)		1.62 (0.45, 5.76)	
**All three**	3/16 (18.8%)	3/18 (16.7%)	1.15 (0.11, 11.7)		0.74 (0.02, 26.8)	

*. Adjusted for age, NIHSS and delay to randomisation (all linear); 2 P for interaction of rtPA/Control with subgroup factor

Supplementary Table 5. **A**. Effect of combinations of imaging signs on SICH: Logistic regression model for SICH with age, NIHSS, time to randomisation, treatment and combinations of old lesions and hyperattenuated artery as predictors. There were 118/3017 patients with SICH. Absolute numbers (%) see 5B below.

**Effect**	**Odds ratio**	**95% confidence limits**
Age (per year increase)	1.00	0.98, 1.02
NIHSS (per point increase)	1.07	1.04, 1,10
Time to randomisation (per hour increase)	0.98	0.83, 1.15
Treatment control vs alteplase	0.15	0.09, 0.26
Old infarct or hyperattenuated artery (but not both) vs neither	1.22	0.78, 1.90
Both old infarct and hyperattenuated artery vs neither	2.98	1.71, 5.16

Supplementary Table 5 B. Absolute excess of SICH events after alteplase per 1000 patients as a function of old lesions and hyperattenuated artery

	**alteplase**		**Control**		
	**N**	**% SICH (95% CI)**	**N**	**% SICH (95% CI)**	**Excess SICH events per 1000 (95% CI)**
**No old infarct or hyperattenuated arteries**	590	4.4 (2.8, 6.1)	605	1.2 (0.3, 2.0)	32 (14, 51)
**Either old infarct or hyperattenuated arteries**	773	6.7 (5.0, 8.5)	800	0.8 (0.2, 1.3)	60 (41, 78)
**Both old infarct and hyperattenuated arteries**	144	16.7 (10.6, 22.8)	105	2.9 (-0.3, 6.0)	138 (69, 207)

Supplementary Table 6A. Effect of combinations of imaging signs on functional outcome: Logistic regression model for odds of six month independent survival (OHS 0-2) with age, NIHSS, time to randomisation, treatment and combinations of large lesions, leukoaraiosis and hyperattenuated artery as predictors. Absolute numbers (%) see Supplementary Table 6B below.

Effect	Odds ratio	95% confidence limits
Age (per year increase)	0.96	(0.96, 0.97)
NIHSS (per point increase)	0.83	(0.82, 0.85)
Time to randomisation (per hour increase)	1.04	(0.96, 1.12)
Treatment control vs alteplase	0.89	(0.75, 1.06)
One out of large lesions, leukoaraiosis, hyperattenuated artery vs None	0.72	(0.59, 0.88)
Two out of large lesions, leukoaraiosis, hyperattenuated artery vs None	0.43	(0.31, 0.58)
All of large lesions, leukoaraiosis, hyperattenuated artery vs None	0.24	(0.12, 0.49)

Supplementary Table 6 B Absolute excess primary outcome (OHS 0-2) events after alteplase as a function of large lesion, hyperattenuated artery and leukoaraiosis

	alteplase		Control		
	**N**	**% OHS 0-2 (95% CI)**	**N**	**% OHS 0-2 (95% CI)**	**Excess OHS 0-2 events per 1000 (95% CI)**
**None out of large lesions, leukoaraiosis, hyperattenuated artery**	486	51.2 (46.8, 55.7)	492	51.0 (46.6, 55.4)	2 (-60, 65)
**One out of large lesions, leukoaraiosis, hyperattenuated artery**	703	35.8 (32.3, 39.4)	717	32.2 (28.8, 35.6)	36 (-13, 86)
**Two out of large lesions, leukoaraiosis, hyperattenuated artery**	254	17.3 (12.7, 22.0)	230	17.8 (12.9, 22.8)	-5 (-73, 63)
**All of large lesions, leukoaraiosis, hyperattenuated artery**	64	7.8 (1.2, 14.4)	71	7.0 (1.1, 13.0)	8 (-81, 96)

Note: Percents and their difference are unadjusted; CI for excess events is based on normal approximation to binomial distribution.

Supplementary Figure 1. Associations between early ischemic or pre-existing (structural) signs on baseline imaging and a) increasing age (all P<0.02 except large lesion; increasing atrophy, leukoaraiosis and old infarcts, all P<0.0001), b) increasing NIHSS (all p<0.0001), and c) increasing time to randomisation (no association with early ischemic signs; pre-stroke signs all P<0.0001). See Table 1 main paper for adjusted Odds ratios. 'Hypodensity' = hypoattenuation.

A) Age

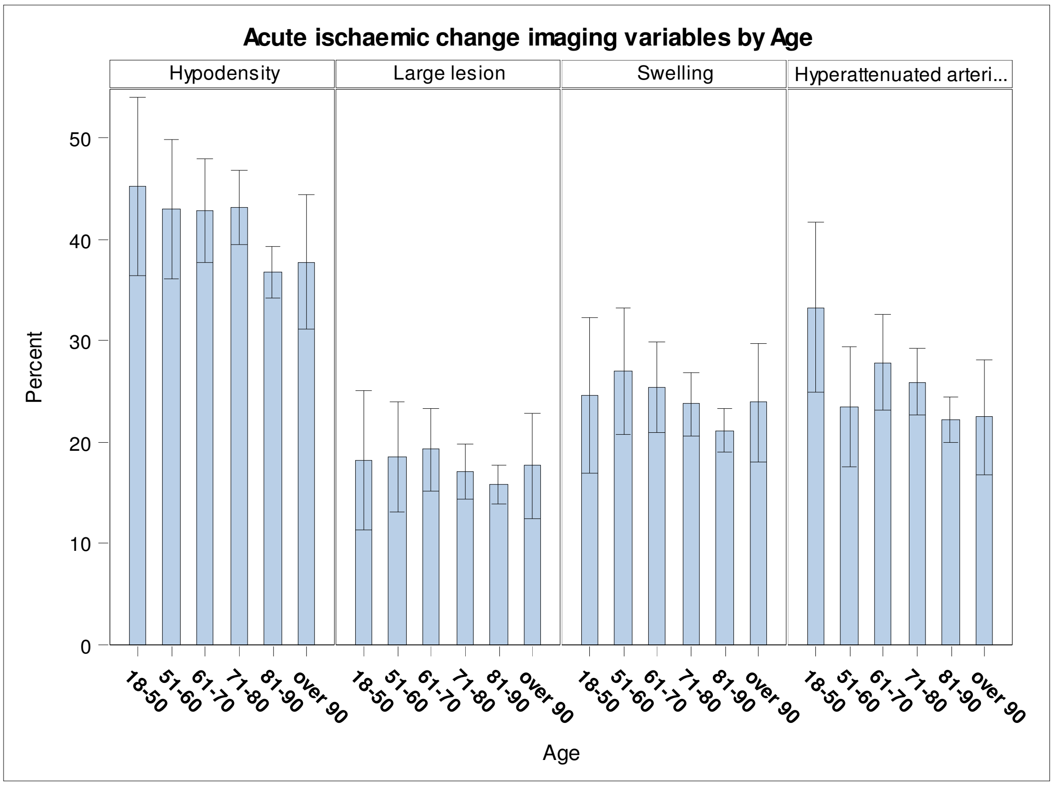

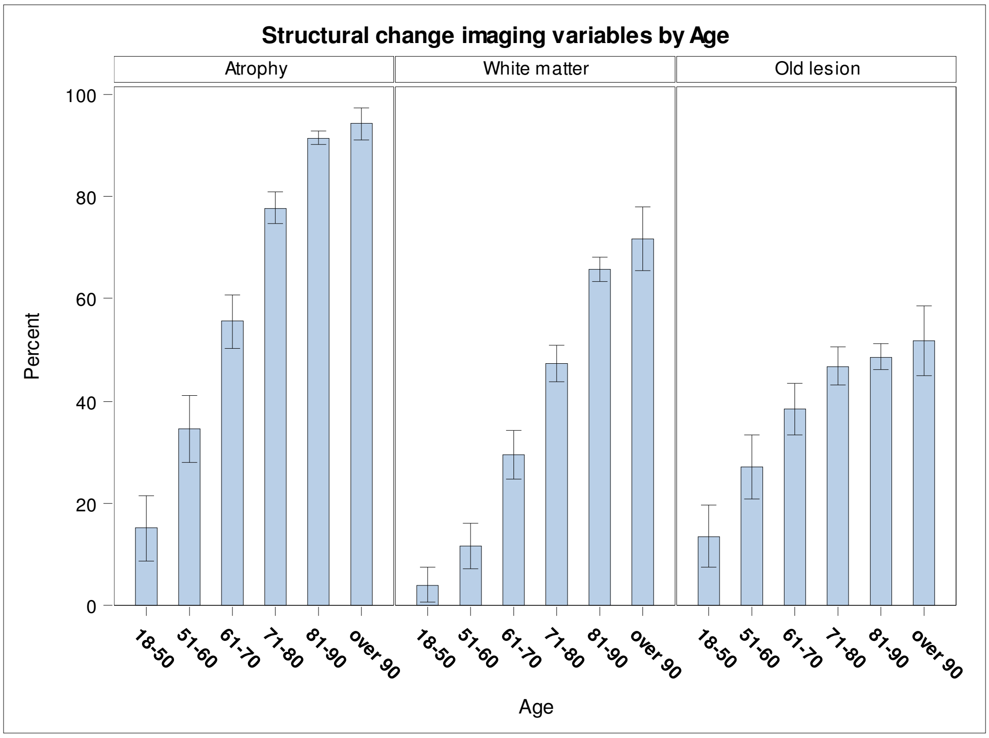

B) NIHSS

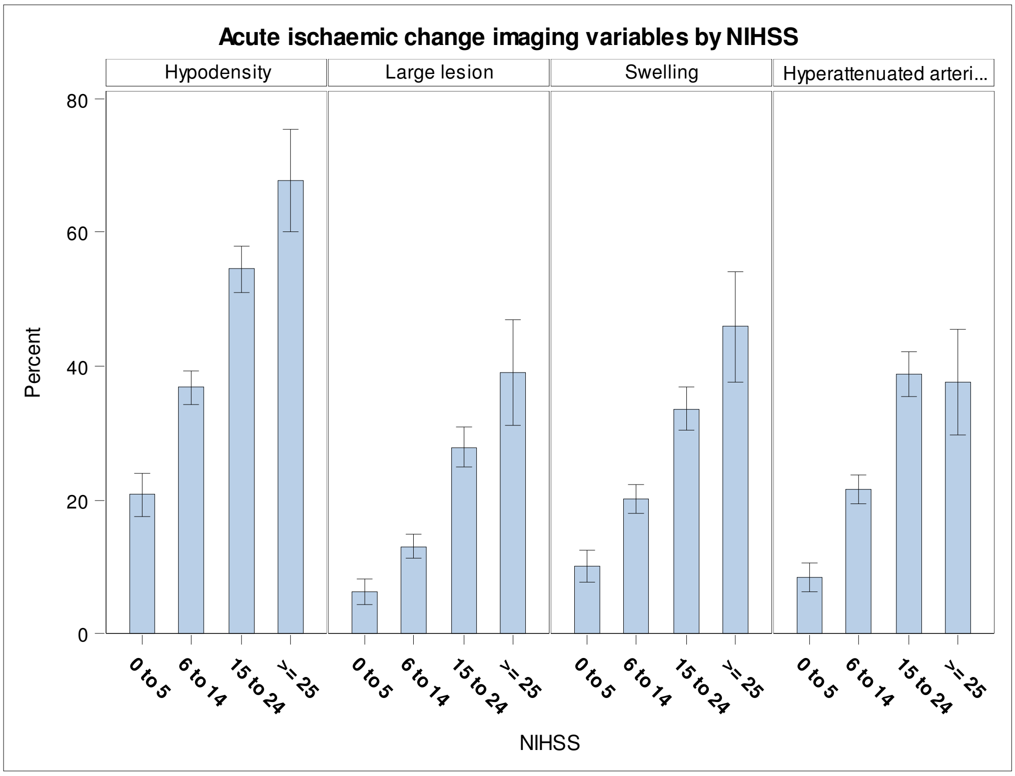
Acute ischaemic change imaging variables by NIHSS
Hypodensity
Large lesion
Swelling
Hyperattenuated arteri...
Percent
0
20
40
60
80
0 to 5
6 to 14
15 to 24
>= 25
NIHSS

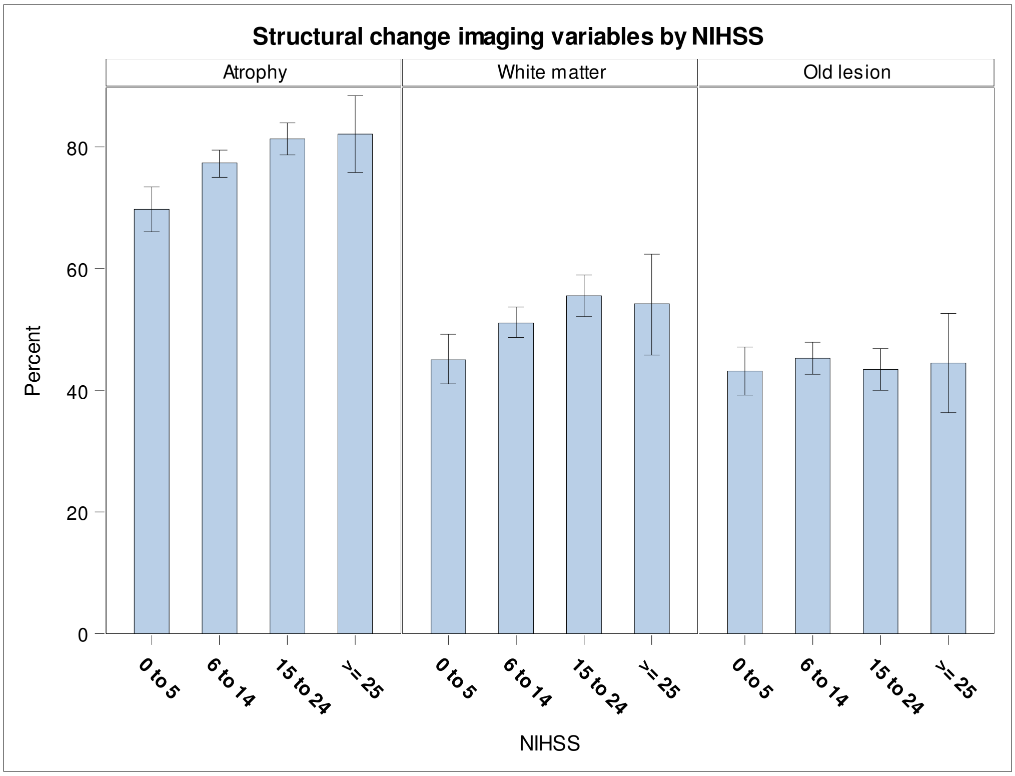
Structural change imaging variables by NIHSS
Atrophy
White matter
Old lesion
Percent
0
20
40
60
80
0 to 5
6 to 14
15 to 24
>= 25
NIHSS

C) Time to randomisation

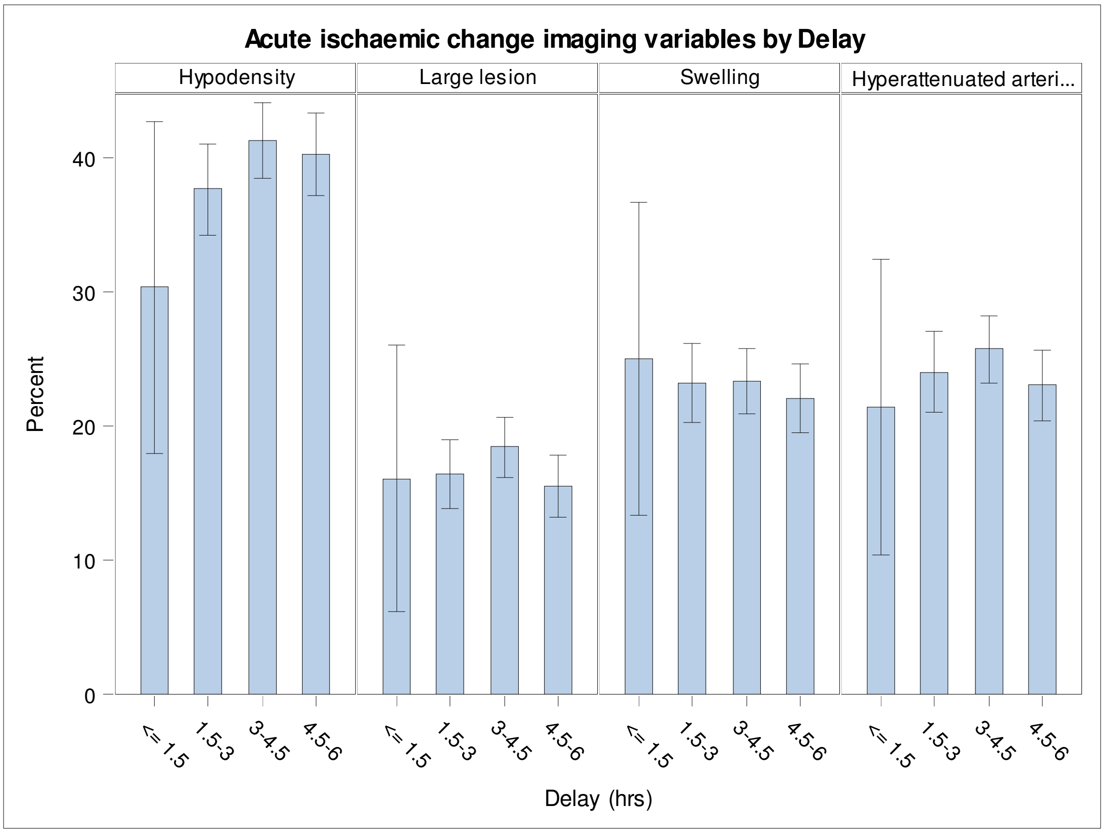

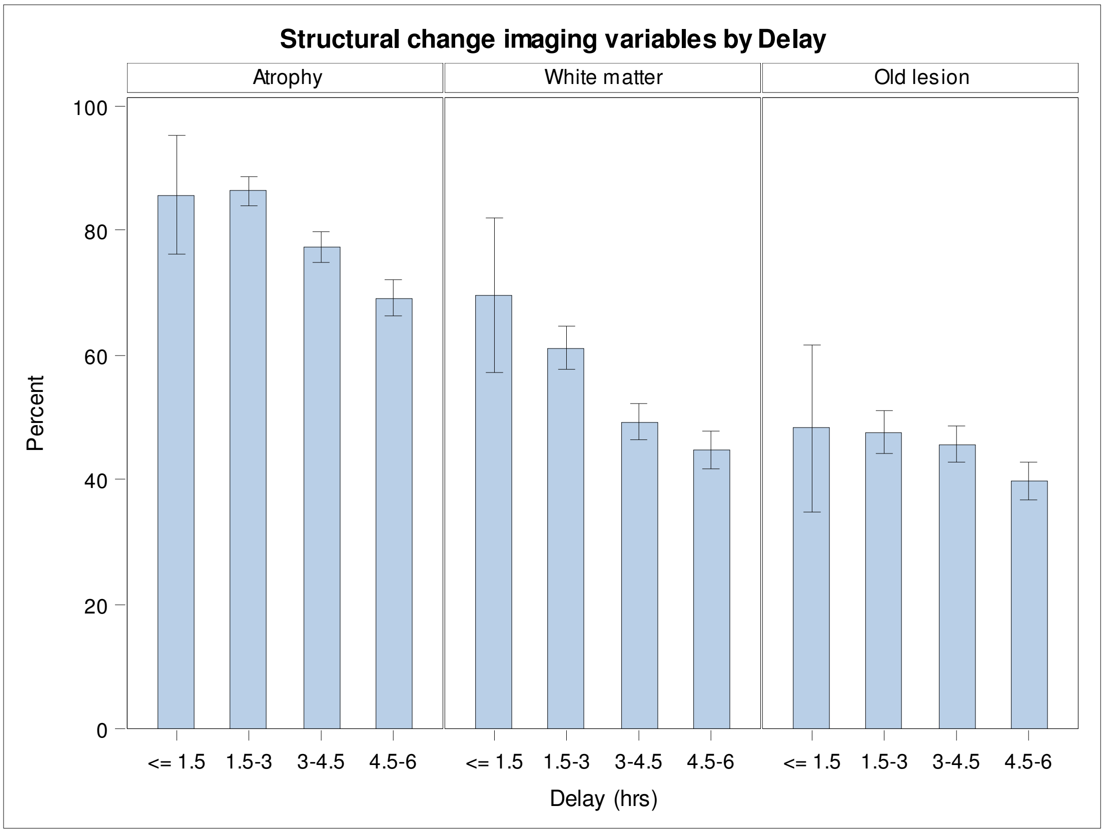

References

1 Wardlaw JM, Sellar RJ. A simple practical classification of cerebral infarcts on CT and its interobserver reliability. *AJNR Am J Neuroradiol* 1994;**15**:1933-9.

2 Wardlaw JM, Farrall AJ, Perry D et al. Factors influencing the detection of early computed tomography signs of cerebral ischemia. An internet-based, international multiobserver study. *Stroke* 2007;**38**:1250-6.

3 Barber PA, Demchuk AM, Zhang J, Buchan AM. Validity and reliability of a quantitative computed tomography score in predicting outcome of hyperacute stroke before thrombolytic therapy. ASPECTS Study Group. Alberta Stroke Programme Early CT Score. *Lancet* 2000;**355**:1670-4.
